# Outcomes of limited fasciectomy, needle fasciotomy and collagenase injection for Dupuytren’s disease: a systematic review and meta-analysis of individual patient data

**DOI:** 10.1177/17531934251338349

**Published:** 2025-05-20

**Authors:** Bente A van den Berge, Hosniya Habibi, Pieter U Dijkstra, Isam Atroshi, Tim RC Davis, Per Jenmalm, Annet van Rijssen, Ruud W Selles, Peter Scherman, Joakim Strömberg, Simon T Skov, Esther Vögelin, Paul MN Werker, Dieuwke C Broekstra

**Affiliations:** 1 Department of Plastic Surgery, University of Groningen, The Netherlands; 2 Center for Rehabilitation, University of Groningen, The Netherlands; 3 Department of Orthopedics Hässleholm-Kristianstad and Department of Clinical Sciences, Lund University, Sweden; 4 Nottingham University Hospitals, UK; 5 Section for Hand and Plastic Surgery, Department of Surgery and Perioperative Science, Umeå University, Sweden; 6 Department of Plastic Surgery, Isala, The Netherlands; 7 Department of Plastic, Reconstructive and Hand Surgery, Erasmus MC – University Medical Centre, The Netherlands; 8 Department of Rehabilitation Medicine, Erasmus MC – University Medical Centre, The Netherlands; 9 Department of Hand Surgery, Skane University Hospital, Malmö, Sweden Department of Translational Medicine – Hand Surgery, Lund University, Sweden; 10 Department of Hand Surgery, University of Gothenburg, Sahlgrenska Academy, Institute for Clinical Sciences, Sweden; 11 Elective Surgery Centre, Silkeborg Regional Hospital, Denmark; 12 Hand Surgery and Surgery of Peripheral Nerves, Inselspital, University of Bern, Switzerland

**Keywords:** Contracture correction, complications, Dupuytren contracture, recurrence, treatment

## Abstract

This systematic review and meta-analysis of individual patient data evaluates the outcomes of treatment for Dupuytren's disease using limited fasciectomy (LF), percutaneous needle fasciotomy (PNF) and collagenase clostridium histolyticum (CCH) injection. A total of 1423 studies were identified, of which 15 met the eligibility criteria for meta-analysis. The postoperative total extension deficit was smaller after LF than after PNF or CCH, but the difference was not clinically relevant. Minor complications were more frequent after CCH than after LF and PNF. The risk of major complications did not differ between the treatments. Recurrence occurred earlier after PNF and CCH than after LF during 36 months of follow-up. Patient-reported outcome measures showed substantial heterogeneity, which precluded meta-analysis. Overall, the clinically relevant contracture correction was comparable between LF, PNF and CCH, but CCH had a higher risk of minor complications and LF had the longest time to recurrence. Treatment decisions should consider the trade-off between complications and recurrence risk.

## Introduction

There is no curative or preventive treatment for Dupuytren’s disease (DD). Treatment is symptomatic by reducing contractures mechanically or enzymatically to improve hand function (Shih and Bayat, 2010). There are several outcomes to consider when choosing the most appropriate treatment for a patient. The most important treatment outcomes are contracture correction, risk of complications, patient-reported hand function and risk of recurrent joint contractures (Karpinski et al., 2020). Although several comparative studies have been conducted on these main treatment outcomes (Van Rijssen et al., 2012; Skov et al., 2017; Strömberg et al., 2018), the results vary between studies and cannot be compared owing to differences in study design, lack of correction for potential confounders in observational studies and- heterogeneity in outcome measures (Karpinski et al., 2020). These factors have made it difficult to perform a meta-analysis in previous systematic reviews (Hirase et al., 2021; Rodrigues et al., 2015; Soreide et al., 2018). The problem of heterogeneity can be partially addressed by performing a meta-analysis of individual patient data (IPD) derived from comparative studies. In an IPD meta-analysis, which is considered the reference standard for systematic reviews (Riley et al., 2010), one operational definition for each desired outcome of interest can be applied to the available data. In this systematic review and IPD meta-analysis, we compared the contracture correction, complications, patient-reported outcome measures (PROMs) and recurrence after treatment for DD with limited fasciectomy (LF), percutaneous needle fasciotomy (PNF) and collagenase clostridium histolyticum (CCH).

## Materials and methods

This systematic review with IPD meta-analysis was registered on PROSPERO (CRD42020199046) and followed the Preferred Reporting Items for a Review and Meta-Analysis guidelines for IPD (Stewart et al., 2015).

### Eligibility criteria

We used a two-stage study selection process. First, we screened titles and abstracts. Eligible studies had to be peer-reviewed, published studies reporting treatment outcomes for LF, PNF and/or CCH. We excluded case reports, reviews, conference abstracts, editorials, studies with unavailable abstracts and studies published before 1 January 2000, as PNF and CCH have only become established approaches in the last 20 years (Foucher et al., 2001; Hurst et al., 2009). There were no language restrictions.

In the second round, included full-text manuscripts were assessed for eligibility criteria for each outcome measure (Online Figure S1). We excluded studies that included only patients treated in the thumb, studies that included patients <18 years of age, and studies for which full text was not available.

### Search strategy and selection process

We searched MEDLINE (via PubMed), Embase, Web of Science and the Cochrane Central Register of Controlled Trials using database-specific search strings on 29 June 2020 and updated the search on 16 March 2022 (Appendix S1). In addition, we checked the reference lists of included studies and related reviews for studies missed by the searches. The first and second author independently assessed titles, abstracts and full-text articles, including studies that met the eligibility criteria for at least one outcome measure. Any reason for study exclusion was recorded. Disagreements and questions about eligibility were discussed with the senior author (PW) until consensus was reached.

### Data collection

From all studies eligible for at least one outcome measure, we documented the type(s) of treatment studied, country/countries of origin, number of study centres, study design, year of study start and end, follow-up time, number of participants, conflict of interest and funding. For all studies that compared LF, PNF and/or CCH, we contacted the corresponding and senior author to request anonymized IPD in an electronic dataset. The completeness and data consistency of each dataset received were assessed. In case of ambiguity or questions about the data, we contacted the author(s). After coding was standardized across all studies, we merged the datasets into a single database. All patients had given informed consent at the time of enrolment in each respective study, and each study had been approved by a medical ethics committee according to the legislation of the country.

### Risk of bias assessment

The first and last author independently assessed the risk of bias of the included studies using the full text and, where available, the study protocol and analysis plan. We assessed the risk of bias for each of the four primary outcomes separately. Randomized controlled trials (RCTs) were assessed using the Cochrane Risk of Bias-2 tool (Sterne et al., 2019), and non-randomized trials were assessed using the Risk of Bias In Non-randomized Studies of Interventions-I tool (Sterne et al., 2016). Any disagreement between assessors was resolved by discussion. We calculated an overall agreement (percentage and linear weighted Cohen’s kappa) based on the ratings for each domain.

### Data handling

#### Contracture correction

We looked at four primary outcomes: contracture correction, complications, patient-reported hand function and recurrence. Contracture correction was defined as correction of the total extension deficit (TED) of the respective finger measured before and between 4 and 12 weeks after treatment. We calculated the preoperative and postoperative TED by summing up metacarpophalangeal (MCP) joint and the proximal interphalangeal (PIP) joint contractures, after correcting any negative values due to hyperextension to zero. Distal interphalangeal joints were not included because distal interphalangeal joint joint contractures were reported in only half of the included studies.

The TED can be measured as total active extension deficit (TAED) or total passive extension deficit (TPED). It has been previously reported that active extension deficit (AED) and passive extension deficit (PED) measurements can differ significantly (Nordenskjöld et al., 2018), but it is unknown whether the degree of contracture correction also varies between AED and PED measurements. Therefore, we evaluated the distribution of contracture correction measured as TAED and TPED in our study population. If the medians and variances of these two methods were similar, we would include them equally in the analysis. If the median TAED and TPED values differed by more than the maximum measurement error in one finger of 15° (Broekstra et al., 2015), we would perform a sensitivity analysis without joint contractures reported as TAED.

#### Complications

All reported complications measured between 1 week and 3 months after treatment were classified as none, minor (such as oedema, blister and haematoma), or major (such as arterial-, neural-, tendon- or ligamentous injury and infection) (Online Table S1). If a patient had both a minor and a major complication, they were classified as a major complication. As the number of complications recorded varied between studies, we recorded the maximum possible complications for each patient.

#### Patient-reported hand function

Patient-reported hand function was measured using PROMs validated for DD. The PROM scores were to be collected both before and after treatment. As there are several PROMs available for use in DD (Bradet-Levesque et al., 2022), we expected that the instruments used would differ between studies. Therefore, we aimed to standardize the different PROM scores in order to perform a single meta-analysis on all PROM scores available in the IPD. The inclusion of different PROMs in a meta-analysis is only appropriate if the correlation between the PROMs is high (Higgins et al., 2022). Therefore, we determined the correlation between the instruments reported in the literature. If we considered the correlation to be insufficient (*r* < 0.8), we planned to analyse the scores of each PROM separately, provided that at least three studies used the same PROM and that either IPD or aggregated results were available.

#### Recurrence

Recurrence was defined as a ≥30° increase in TED in the treated digit compared with the postoperative measurement measured between 4 and 12 weeks after treatment. Patients who received repeat treatment of the same digit during follow-up were also classified as having a recurrence. Most comparative studies use periodic clinical examinations (e.g. at 12 months and 24 months) to assess recurrence. As a result, the time to the event is not known exactly, because it is not known whether the recurrence occurred, for example, after 13 months or 23 months (interval censoring). We have taken this uncertainty into account in our analysis.

### Data analysis

We used a one step-approach, which involves a simultaneous analysis of IPD retrieved from eligible studies. Some studies included multiple fingers per patient. In these cases, we randomly selected one finger using the *dplyr* package in R (R Core Team, 2022; Wickham et al., 2022).

#### Contracture correction

We used a linear mixed model with postoperative TED as the outcome. In addition to treatment (LF, PNF or CCH), we included age, preoperative TED and whether it was a primary or repeat treatment as independent variables in the model to account for potential confounding effects. As the residuals in our data were not normally distributed, we performed a square root transformation on the postoperative TED. This resulted in a normal distribution of the residuals. Results were reported as regression coefficients, with corresponding 95% confidence intervals (CIs). As transformed coefficients are difficult to interpret, we also reported the estimated values of the postoperative TED for each treatment.

#### Complications

We used a multinomial regression analysis with complication (none, minor or major) as the outcome, and treatment, preoperative TED, age, primary or repeat treatment and the number of maximum potential complications (based on the number of complications recorded in the study) as independent variables in our model. Results were reported as odds ratios with corresponding 95% CIs.

#### Patient-reported hand function

We chose a linear mixed model, with the postoperative PROM score(s) as the outcome, adding treatment, preoperative PROM score, age and primary or recurrent disease as explanatory variables to the model and a random intercept to our model for the study.

#### Recurrence

We calculated the number and percentage of recurrences and analysed the time to recurrence for each treatment. As not all studies measured recurrence at the same time points, we applied survival analysis taking into account follow-up times. In addition, to account for interval censoring, we used a parametric interval-censored survival model with a log–normal distribution, which was found to have the best model fit. We included treatment, age, sex and primary or recurrent disease as potential confounders. Patients for whom it was unknown whether they had a recurrence were excluded. Results were reported as regression coefficients and corresponding time ratios, which quantify the relative difference in time to recurrence.

All studies were controlled for the potential clustering by adding a random intercept or stratum for the original study, the risk of bias, and the study design.

## Results

### Study selection and characteristics

**Figure 1.**
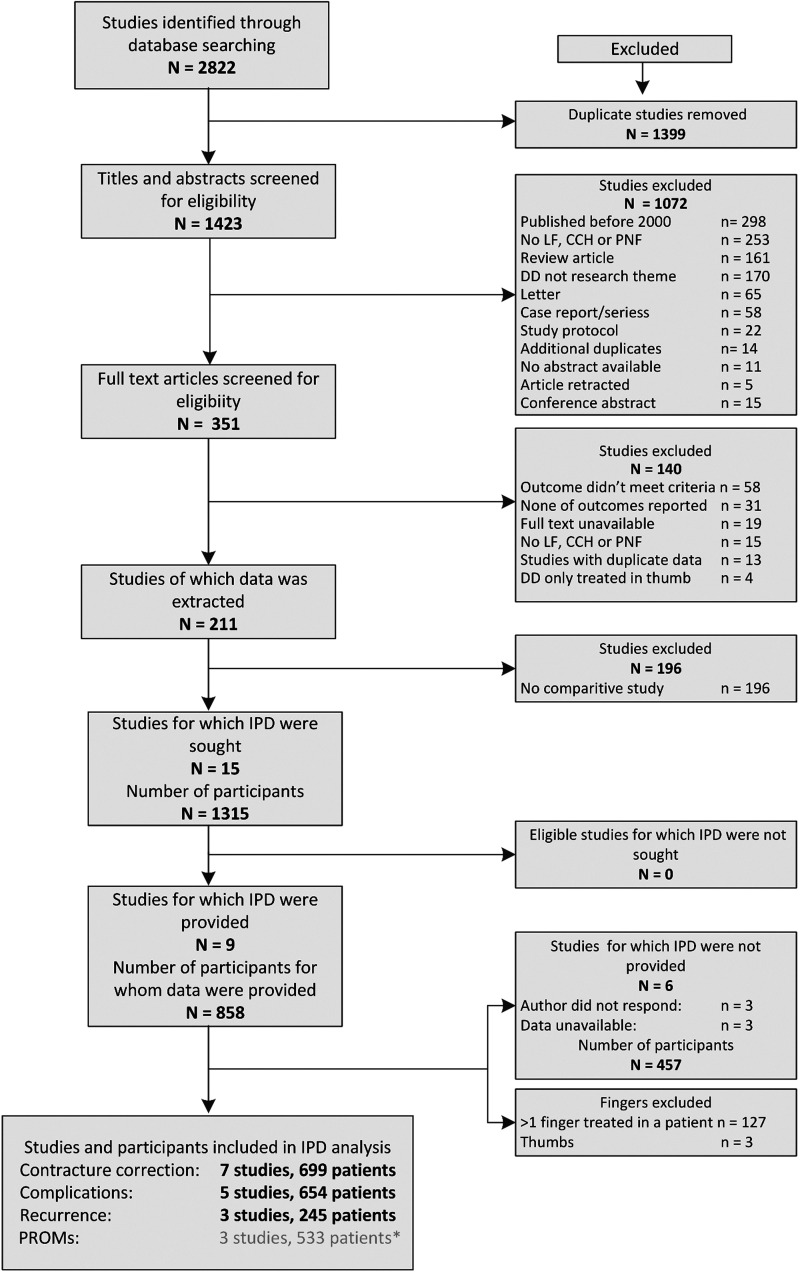
Study selection process. * We were unable to perform a meta-analysis for this outcome. IPD, Individual patient data; DD, Dupuytren’s disease; CCH, collagenase clostridium histolyticum; LF, limited fasciectomy; PNF, percutaneous needle fasciotomy.

We identified 2822 papers, of which 211 studies met the eligibility criteria for at least one outcome (Figure 1, Online Table S2). Fifteen studies (1315 patients), six RCTs and nine cohort studies were eligible for inclusion in the meta-analysis (Figure 1). We obtained IPD from nine studies (858 patients, 65%) (Table 1). For most of the RCTs, we had some concerns about the risk of bias. We rated all cohort studies as having some concerns or a high risk of bias (Online Table S3). There was 63% agreement (weighted Cohen’s kappa 0.38, 95% CI 0.28; 0.47). We were unable to perform sensitivity analyses after excluding all studies with a high risk of bias, as this would have left too few studies per outcome measure to analyse.

**Table 1.** Study designs and patient characteristics of 15 eligible studies

	Country	Intervention	Outcomes	Design	*N*	*N*/group	Age (SD)	Sex ♂	FU	Prim./rec.	IPD
Vollbach 2013	DEU	LF vs. CCH	A, C, P, R	Cohort (s)	27	13/14	61 (3)	81%	12 m	NR	No
Atroshi 2014	SWE	LF vs. CCH	A, C	Cohort (s)	32	16/16	70 (5)	75%	2 m	Unclear	Yes
Wei 2015	Unclear	LF vs. CCH	C	Cohort (s)	37	19/18	66 (9)	76%	>24 m	NR	No
Zhou 2015	NLD	LF vs. CCH	A, C, P	Cohort (m)	218	114*/104	62 (9)	80%	3 m	Both	Yes
Wehrli 2016	CHE	LF vs. CCH	P	Cohort (s)	55	11/44	65 (9)	81%	12 m	NR	No
Leclère 2018	CHE	LF vs. CCH	A, C, P	Cohort (s)	57	14/43	64 (9)	85%	24 m	NR	Yes
Hensler 2020	CHE	LF vs. CCH	P	Cohort (s)	231	NR	67 (9)	NR	12 m	NR	No
Ribak 2013	BRA	LF vs. PNF	A, C, R	Cohort (s)	33	17/16	NR	91%	12 m	Primary	No
Zhou 2016	NLD	LF vs. PNF	A, C, P	Cohort (s)	193	114*/79	65 (8)	70%	3 m	Both	Yes
Davis 2020	GBR	LF vs. PNF	A, C, P	RCT (m)	71	33/38	66 (8)	76%	6 m	Primary	Yes
van Rijssen 2006; 2012	NLD	LF vs. PNF	A, C, R	RCT (s)	112	51/61	63 (NR)	83%	60 m	Primary	Yes
Skov et al. 2017	DNK	PNF vs. CCH	A, C, R	RCT (s)	50	22/28	64 (9)	77%	24 m	Primary	Yes
Abe 2020	JPN	PNF vs. CCH	A, C, P, R	RCT (s)	72	36/36	68 (NR)	93%	36 m	Primary	No
Scherman 2016; 2018	SWE	PNF vs. CCH	A, C, P, R	RCT (m)	83	44/39	67 (NR)	77%	36 m	Primary	Yes
Stromberg 2016; 2018	SWE	PNF vs. CCH	C, P	RCT (s)	156	78/78	67 (9)	85%	24 m	Primary	Yes

*The patients treated with LF are the same patients in both studies from Zhou. We included them only once in the analysis. For studies from which IPD was obtained, *N* number of patients represents the number of patients included in the IPD analysis. For some studies, the number of patients for which we retrieved IPD was higher than the number of patients the authors included in their analyses: Zhou et al. 2015 performed propensity score matching, including only 132 of the 218 patients for which we retrieved IPD. The IPD of Leclère et al. contained 57 patients, while they included 52 patients in their analysis. From the studies of Leclère, Atroshi and Van Rijssen, IPD on complications were unavailable. Countries are abbreviated according to the ISO 3166 country codes. For the study of Wei et al., it was unclear whether the study took place in the USA or Malaysia. A, Angular contracture correction; C, complications; P, PROMs; R, recurrence; (s), single centre; (m), multi-centre; NR, not reported; CCH, collagenase clostridium histolyticum; LF, limited fasciectomy; PNF, percutaneous needle fasciotomy; RCT, randomized controlled trial; prim., primary disease; rec., recurrent disease; IPD, individual patient data retrieved; FU, follow-up time (months).

### Contracture correction

Eight studies (702 patients) met the eligibility criteria for contracture correction. We excluded three patients because their thumb was the treated digit. Median contracture correction measured as TPED (40°, interquartile range (IQR) 30, 58) was comparable with contracture correction measured as TAED (38°, IQR 22, 55). Therefore, we included TAED and TPED in one analysis. The baseline characteristics of the patients included in each analysis are shown in Table 2.

**Table 2.** Baseline characteristics of patients included in the contracture correction, complications and recurrence analysis

Contracture correction	CCH	LF	PNF
*N*	226	228	245
Age, median [IQR]	65 [59, 69]	64 [58, 69]	67 [59, 71]
Sex (m), *n* (%)	185 (82)	185 (81)	189 (77)
Preoperative extension deficit			
MCP-joint contracture, *n* (%)	166 (73)	151 (66)	196 (80)
MCP-joint contracture, median [IQR]	43° [26, 60]	40° [23, 54]	45° [31, 60]
PIP-joint contracture, *n* (%)	148 (65)	176 (77)	171 (70)
PIP-joint, median [IQR]	37° [20, 56]	44° [26, 60]	30° [15, 50]
TED, median [IQR]	55° [40, 70]	56° [40, 75]	60° [40, 78]
*Missing TED, n (%)*	2 (0.9)	6 (2.6)	10 (4.2)
Postoperative extension deficit			
MCP-joint contracture, median [IQR]	0° [0, 14]	0° [0, 5]	5° [0, 15]
PIP-joint contracture, median [IQR]	12° [5, 28]	11° [2, 24]	15° [8, 25]
TED, median [IQR]	10° [0, 29]	10° [0, 25]	15° [8, 30]
*Missing TED, n (%)*	15 (7)	16 (7)	11 (5)
Correction TED, median [IQR]	40° [25, 55]	40° [30, 59]	40° [28, 53]
*Missing, n (%)*	14 (6)	17 (8	14 (6)
Previous treatment, *n* (%)			
Yes	27 (12)	43 (19)	15 (6)
No	157 (69)	171 (75)	230 (94)
*Missing*	42 (18)	14 (6)	0 (0)
Treated digit, *n* (%)			
Index finger	1 (0)	1 (0)	4 (2)
Middle finger	15 (7)	18 (8)	19 (8)
Ring finger	75 (33)	58 (25)	79 (32)
Little finger	119 (53)	135 (59)	143 (58)
*Missing*	16 (7)	16 (7)	0 (0)
Complications	CCH	LF	PNF
*N*	244	147	263
Age, median [IQR]	64 [59, 69]	64 [57, 69]	67 [62, 72]
Sex (m), *n* (%)	200 (82)	119 (81)	205 (78)
Complications, *n* (%)			
None	78 (32)	114 (78)	222 (84)
Mild	157 (64)	26 (18)	33 (13)
*Missing*	1 (0)	6 (4)	2 (1)
Serious	9 (4)	7 (5)	8 (3)
*Missing*	79 (32)	6 (4)	80 (30)
Previous treatment			
Yes	26 (11)	43 (29)	15 (6)
No	218 (89)	104 (71)	248 (94)
Preoperative TED, median [IQR]	52° [38, 70]	57° [43, 75]	56° [40, 70]
Number registered, median [IQR]	23 [5, 23]	23 [23, 23]	8 [5, 24]
Recurrence	CCH	LF	PNF
*N*	66	51	128
Age, median [IQR]	67 [61, 71]	63 [58, 69]	67 [59, 72]
Sex (m), *n* (%)	56 (85)	42 (82)	108 (84)
Recurrence, *n* (%)	27 (41)	5 (10)	42 (33)
*Missing*	3 (5)	9 (18)	15 (12)
Follow-up in months, median [IQR]	25 [24, 36]	60 [36, 60]	24 [15, 36]

TED, Total extension deficit; IQR, interquartile range; MCP, metacarpophalangeal; PIP, proximal interphalangeal; CCH, collagenase clostridium histolyticum; LF, limited fasciectomy; PNF, percutaneous needle; *N*, the number of patients included in each analysis.

**Figure 2.**
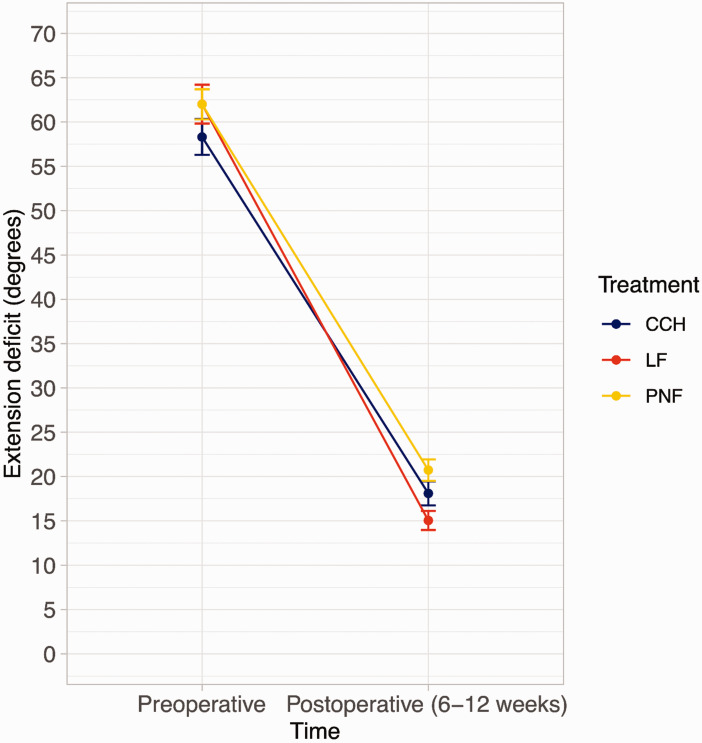
Preoperative and postoperative mean and 95% confidence intervals of the total extension deficit (metacarpophalangeal (MCP) joint + proximal interphalangeal (PIP) joint contractures), measured before and after treatment with CCH, LF or PNF, uncontrolled for confounding factors. CCH, Collagenase clostridium histolyticum; LF, limited fasciectomy; PNF, percutaneous needle fasciotomy.

The results from our model showed that patients treated with LF had a significantly smaller postoperative TED than patients treated with CCH. In addition, the postoperative TED was smaller after CCH than after PNF (Figure 2 and Table 3). The estimated postoperative TED of the average patient treated for primary disease was 5° after LF, 8° after CCH, and 10° after PNF. For recurrent disease, the estimated postoperative TED was 10° after LF, 14° after CCH and 17° after PNF (Online Table S4 and Online Figure S2). Treatment for recurrent disease, larger preoperative TED, risk of bias (low or some concerns) and study design (cohort study) were significantly associated with a larger postoperative TED (Online Table S5).

**Table 3.** Results of the linear mixed effects model on the correction of contracture, the parametric interval-censored survival analysis for time to recurrence, and the multinomial regression analysis for complications after treatment with LF, PNF and CCH. Coefficients are corrected for the influence of confounders.

	Coefficient	95% CI	*p*-Value	Interpretation
Contracture correction (square root transformed)				
PNF vs. LF	0.97	0.59; 1.36	**<0.001** *	PNF resulted in a larger postoperative TED than LF
CCH vs. PNF	−0.45	−0.86; −0.05	**0.028** *	CCH resulted in a smaller postoperative TED than PNF
CCH vs. LF	0.52	0.93; 0.10	**0.015** *	CCH resulted in a larger postoperative TED than LF
Complications				
*Mild*				
PNF vs. LF	−0.55	−1.21; 0.10	0.099	No significant difference in mild complications
CCH vs. PNF	3.06	2.51; 3.61	**<0.001** *	The odds of a mild complication is 21.4 (95% CI 12.4; 37.2) times higher when treated with CCH compared with PNF
CCH vs. LF	2.51	1.92; 3.11	**<0.001** *	The odds of a mild complication is 12.4 (95% CI 6.8; 22.5) times higher when treated with CCH compared with LF
*Serious*				
PNF vs. LF	−1.31	−2.88; 0.26	0.101	No significant difference in serious complications
CCH vs. PNF	0.97	−2.04; 0.09	0.072	No significant difference in serious complications
CCH vs. LF	−0.34	−1.93; 1.25	0.675	No significant difference in serious complications
Recurrence				
PNF vs. LF	−1.88	0.70; 3.06	**0.002** *	Recurrence occurred 5.8 (95% CI 1.7; 20.0) times earlier in PNF than in LF
CCH vs. PNF	−0.13	−0.52; 0.27	0.532	No significant difference in time to recurrence
CCH vs. LF	−1.75	−2.99; −0.51	**0.006** *	Recurrence occurred 6.5 (95% CI 2.0; 21.3) times earlier in CCH than in LF

In the contracture correction analysis, the estimates represent the regression coefficients in number of degrees of joint contracture after square root transformation. The results are made more insightful by reporting the odds ratios (complications) and time ratios (recurrence analysis) in the column ‘Interpretation’. Both are calculated by exponentiating the regression coefficient.

Bold *p*-value indicates statistical significance

* Statistically significant effect. CI, Confidence interval; TED, total extension deficit; CCH, collagenase clostridium histolyticum; LF, limited fasciectomy; PNF, percutaneous needle fasciotomy.

### Complications

**Figure 3.**
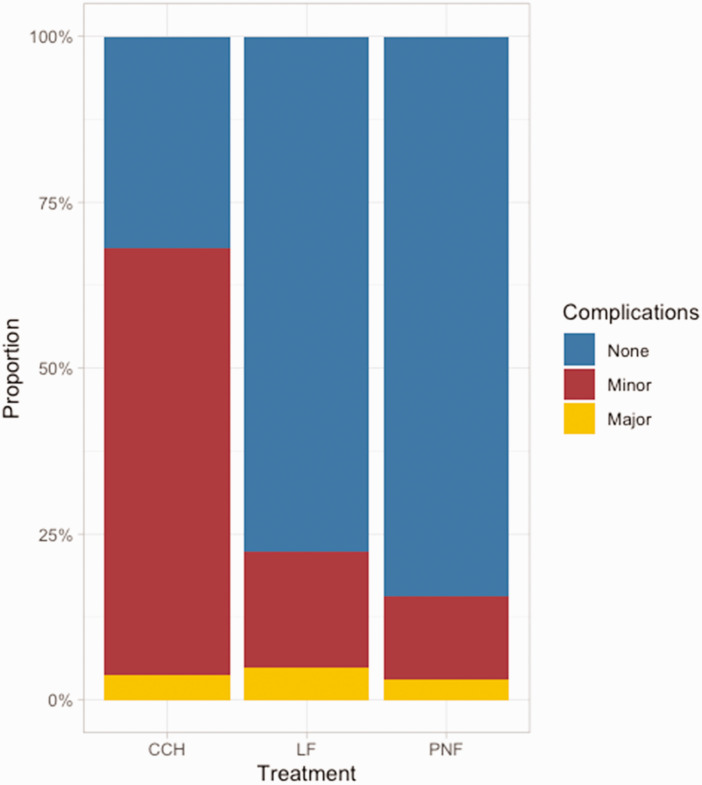
Proportion of patients with no, minor, and major complications, stratified by treatment, uncontrolled for confounders. CCH, Collagenase clostridium histolyticum; LF, limited fasciectomy; PNF, percutaneous needle fasciotomy.

Six studies (654 patients) met the eligibility criteria for complications. Most patients treated with LF (78%) or PNF (84%) had no complications, whereas most patients treated with CCH had one or more minor complications (64%). The number of major complications was similar between the groups (Table 2, Figure 3, Online Table S6). Patients treated with CCH had a higher risk of a minor complication than patients treated with LF or PNF. The risk of a major complication did not differ significantly between all treatments (Table 3). A larger preoperative TED and a higher number of potential/measured complications increased the risk of minor complications, and treatment for recurrent disease was associated with an increased the risk of major complications (Online Table S7).

### PROMs

The correlation between the three most commonly used PROMs (the Unité Rhumatologique des Affections de la Main, the Michigan Hand Questionnaire and the Disabilities of the Arm, Shoulder, and Hand) ranged from 0.55 to 0.60 (Bradet-Levesque et al., 2022; Higgins et al., 2022), which was too weak to include different PROMs in a meta-analysis. In addition, we did not obtain IPD from at least three studies using the same PROM, nor were aggregated results from at least three studies using the same PROM available. Therefore, we were not able to perform a meta-analysis for PROMs (Online Table S8).

### Recurrence

**Figure 4.**
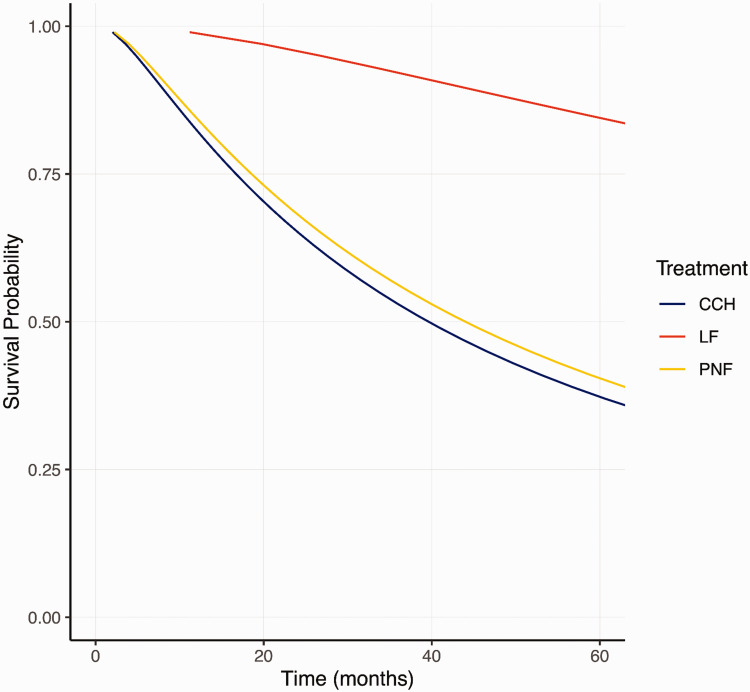
Survival probability after treatment with LF, PNF or CCH. The results presented are not controlled for confounders. CCH, Collagenase clostridium histolyticum; LF, limited fasciectomy; PNF, percutaneous needle fasciotomy.

Three studies (245 patients) met the eligibility criteria for recurrence. Twenty-eight patients (11%) were lost to follow-up. The median follow-up of the remaining patients was 36 months (IQR 24, 36). Recurrence was most common after CCH (41%), followed by PNF (33%) and LF (10%) (Tables 2 and 3). There was no difference in time to recurrence between CCH and PNF (Table 3 and Figure 4). Younger age and female sex were significantly associated with earlier recurrence (Online Table S9).

## Discussion

Our systematic review and meta-analysis of IPD showed that treatment with LF results in a negligibly smaller postoperative TED compared with PNF and CCH. Treatment with LF and PNF is associated with a lower number of minor complications compared with treatment with CCH. Treatment with LF results in a longer time to recurrence compared with PNF and CCH.

Since the completion of our analysis, several recent systematic reviews and meta-analyses with similar aims have been published (Liechti et al., 2024; Nann et al., 2023). These are all aggregated meta-analyses that are influenced by the quality of reporting of the underlying studies. This has several limitations, in particular variation in outcome definitions and the inability to adjust for confounders (Koopman et al., 2007). For example, Nann et al. (2023) did not pre-define key outcomes such as recurrence, which introduces variability that cannot be properly accounted for in aggregated meta-analyses. In addition, an aggregated data meta-analysis cannot adjust for confounders such as preoperative TED, which is crucial for a fair comparison between studies with different treatment strategies (e.g. treatment of PIP or MCP joints singularly or together). In contrast, our meta-analysis of IPD used consistent outcome definitions and adjusted for critical confounders, which provides stronger evidence of differences in outcomes for Dupuytren disease than previous reports.

Of the large number of studies published on treatment outcomes of LF, PNF or CCH, the majority have either a self-controlled study design (*n* = 70, 33%), in which differences in treatment outcomes before and after treatment in a single population were analysed, or an uncontrolled study design (*n* = 67, 32%), in which treatment outcomes in a single population were described. Both designs are considered as having low level of evidence (Burns et al., 2011). Relatively few trials had a prospective design comparing two treatments. We retrieved data from nine out of 15 eligible studies, representing 858 (65%) of 1315 patients, which is an acceptable retrieval rate compared with other IPD analyses (Nevitt et al., 2017).

Postoperative TED was significantly smaller after treatment with LF compared with PNF and CCH and after CCH compared with PNF. However, the largest difference in estimated postoperative TED was 5° (5° after LF and 10° after PNF) after primary treatment and 7° (10° after LF and 17° after PNF) after treatment for recurrent disease. The minimal clinically important difference in contracture, which reflects the change in contracture that is meaningful to the patient, was estimated to be 14° (95% CI 12, 15) (Witthaut et al., 2011). Furthermore, the differences of 5° and 7° do not exceed the maximum measurement error in a finger of 15° (Broekstra et al., 2015). Therefore, although statistically significant, the differences in contracture correction are unlikely to be perceived as relevant by the patients.

Our results showed that the proportion of patients with a major complication (e.g. nerve, tendon or artery damage) was highest after LF, but there was no significant difference between treatments. However, the number of minor complications was significantly higher in patients treated with CCH. The most common minor complications were bruising (63%), oedema (50%) and pain or local discomfort (48%). Collagenase clostridium histolyticum causes a chemical and immunological response, which may be responsible for the higher number of minor complications such as swelling, pruritus, oedema and lymphadenopathy (Hurst et al., 2009). In addition, the early studies involving CCH were drug trials, which are subject to strict regulations on the recording and reporting of adverse events (Hurst et al., 2009). More adverse events were reported in these studies than in studies of PNF and LF (Hurst et al., 2009; van Rijssen et al., 2006). However, it is unlikely that the number of reported complications is the main reason for the large difference in the number of minor complications, as we adjusted our analysis for the number of reported complications per protocol.

As both IPD and aggregated results were only available for one or two studies per PROM, we were unable to perform a meta-analysis of either IPD or aggregated data with this outcome. We therefore conclude that there is great heterogeneity in the use of PROMs in DD research. Until there is consensus on the most appropriate PROM for DD, it is impossible to compare treatments for this outcome.

We defined recurrence as an increase in TPED of ≥30° compared with 4–12 weeks after treatment, as the IPD did not always specify which finger joints were treated. Therefore, measuring recurrence as an increase of ≥20° in one joint (Kan et al., 2017) could have led to misclassification of recurrence, so we did not use this definition. The time to recurrence after PNF and CCH was shorter than that after treatment with LF, a known disadvantage of these treatments. Percutaneous needle fasciotomy and CCH can be effectively and safely applied repeatedly for recurrent disease (Bear et al., 2017; van Rijssen and Werker, 2012). In addition, the time to return to work time after LF is reported to be 2–5 weeks, compared with several days after PNF and CCH (Blake et al., 2021; Naam, 2013). Furthermore, the treatment costs of LF are higher than those for PNF and CCH (Chen et al., 2011). However, the current evidence on the cost-effectiveness of these treatments is limited (Dritsaki et al., 2018). Overall, recurrence rates should not be the only treatment outcome presented during shared decision-making for a particular treatment. As CCH has been withdrawn from markets outside the US in 2019, this treatment is only available in the US, where it is still widely used (Yuksel et al., 2022).

We assessed the risk of bias in six RCTs. Sixteen out of 19 outcomes had a moderate risk of bias, and two outcomes had a high risk of bias because the proportion of missing values differed significantly between the two groups, which was likely to be related to both the treatment and the primary outcome. The risk of bias assessed in all seven cohort studies was moderate for five out of 16 outcomes and high for the remaining 11 outcomes, because the authors did not consider one or more potential confounders or did not assess their influence. It is also noteworthy that little is reported about missing values and how they were handled. We analysed the data using a mixed model, which is good at dealing with missing values (Pinheiro and Bates, 2000), and we adjusted our model for several important confounders. This allowed us to estimate the effect of the interventions as accurately as possible. We also planned to perform a sensitivity analysis by repeating the analysis after excluding trials with a high risk of bias. However, this would have left too few trials per outcome measure to analyse. Therefore, we controlled our analyses for risk of bias, which showed a significant effect on postoperative TED, which negatively affected the robustness of our findings. For the outcome complications, the risk of bias was moderate for all studies, meaning that its effect on the robustness of our findings is unclear. Risk of bias was not associated with time to recurrence.

We were able to improve consistency across studies by using IPD from the majority of eligible RCTs and cohort studies and by using a single definition of the most important outcomes of treatment for DD. We were also able to correct for the influence of confounders.

This analysis has limitations. We initially aimed to classify complications according to the International Consortium for Health Outcome Measures classification of complications in hand and wrist conditions (Wouters et al., 2021), modified and derived from the Clavien–Dindo classification (Dindo et al., 2004), but these data were not available, and we could not apply this classification retrospectively. Another limitation was the inclusion of cohort studies and randomized trials. We would have preferred to include only RCTs, but this would have resulted in too few trials to perform a meta-analysis. Our analysis showed that postoperative TED was statistically significantly greater in cohort studies than in RCTs, but the difference is clinically irrelevant. For the outcome of recurrence, all studies were RCTs, so the effect on the robustness of our findings is unclear. There was no effect of study design on complications.

Contracture correction with LF was better than with PNF and CCH, but the differences in postoperative TED were small and not clinically relevant. Treatment with LF is associated with a longer time to recurrence, whereas treatment with CCH is associated with a higher risk of minor complications. To reduce heterogeneity in the definition of surgical outcomes within DD, future studies should prospectively register complications and reach consensus on the most appropriate PROM to be used in DD.

## Supplemental Material

sj-pdf-1-jhs-10.1177_17531934251338349 - Supplemental material for Outcomes of limited fasciectomy, needle fasciotomy and collagenase injection for Dupuytren’s disease: a systematic review and meta-analysis of individual patient data Supplemental material, sj-pdf-1-jhs-10.1177_17531934251338349 for Outcomes of limited fasciectomy, needle fasciotomy and collagenase injection for Dupuytren’s disease: a systematic review and meta-analysis of individual patient data by Bente A van den Berge, Hosniya Habibi, Pieter U Dijkstra, Isam Atroshi, Tim RC Davis, Per Jenmalm, Annet van Rijssen, Ruud W Selles, Peter Scherman, Joakim Strömberg, Simon T Skov, Esther Vögelin, Paul MN Werker and Dieuwke C Broekstra in Journal of Hand Surgery (European Volume)
