# Supplementary material for: Outcomes of limited fasciectomy, needle fasciotomy and collagenase injection for Dupuytren’s disease: a systematic review and meta-analysis of individual patient data
Source: J Hand Surg Eur Vol. 2025 May 20;50(7):878–90. doi: 10.1177/17531934251338349 (PMC12149465; doi:10.1177/17531934251338349)
Supplement: sj-pdf-1-jhs-10.1177_17531934251338349 - Supplemental material for Outcomes of limited fasciectomy, needle fasciotomy and collagenase injection for Dupuytren’s disease: a systematic review and meta-analysis of individual patient data [file sj-pdf-1-jhs-10.1177_17531934251338349.pdf]

Supplementary Material

Title: Outcomes of limited fasciectomy, needle fasciotomy and collagenase in Dupuytren's disease - a systematic review and meta-analysis of individual patient data

B.A. van den Berge et al.

Index

Appendix

Appendix 1 *pag. 2*

Appendix 2

Supplementary Figures

Online Figure 1 *pag. 3*

Online Figure 2 *Pag. 4*

Supplementary Tables

Online Table 1 *pag. 5*

Online Table 2 *pag. 6-11*

Online Table 3 *pag. 12*

Online Table 4 *pag. 13*

Online Table 5 *pag. 14*

Online Table 6 *pag. 15*

Online Table 7 *pag. 16*

Online Table 8 *pag. 17*

Online Table 9 *pag. 18*

Appendices

Appendix 1. Search strategies for MEDLINE, Embase, Web of science, and central

Search strategy<br><br>First search: June 30 2020<br><br>Search update: March 16 2022
Database 1 name: MEDLINE
Search strategy:<br><br>(“Dupuytren Contracture”[MeSH] OR Dupuytren*[tiab] OR fibromatos*[tiab] OR fibroproliferative condition*[tiab] OR fibroproliferative disease*[tiab])<br>AND<br>((Collagenases [MeSH] OR collagenase[tiab] OR CCH[tiab] OR "xiapex" [Supplementary Concept] OR “xiapex”[tiab] OR “xiaflex”[tiab] OR enzymatic[tiab])<br>OR<br>(Fasciotomy[Mesh] OR fasciotom*[tiab] OR aponeurotom*[tiab] OR needle release[tiab] OR PNF[tiab] OR minimally invasive[tiab])<br>OR<br>(fasciect*[tiab] OR aponeurectom*[tiab]))
Database 2 name: Embase
Search strategy:<br><br>('dupuytren contracture'/exp OR 'dupuytren contracture' OR 'dupuytren':ab,ti OR 'fibromatos*':ab,ti OR 'fibroproliferative condition*':ab,ti OR 'fibroproliferative disease*':ab,ti)<br>AND<br>(('collagenase'/exp OR 'collagenase' OR 'collagenase':ab,ti OR 'cch':ab,ti OR 'xiapex':ab,ti OR 'xiaflex':ab,ti OR 'enzymatic':ab,ti)<br>OR<br>('fasciotomy'/exp OR 'fasciotomy' OR 'fasciotom*':ab,ti OR 'aponeurotom*':ab,ti OR 'needle release':ab,ti OR 'pnf':ab,ti OR 'minimally invasive':ab,ti)<br>OR<br>('fasciectomy'/exp OR 'fasciectomy' OR 'aponeurectomy'/exp OR 'aponeurectomy' OR 'fasciectom*':ab,ti OR 'aponeurectom*':ab,ti))
Database 3 name: Web of Science
Search strategy:<br><br>(TS=(dupuytren* OR fibromatos* OR fibroproliferative condition* OR fibroproliferative disease*)<br>AND<br>(TS=(collagenas* OR CCH OR Xiapex OR Xiaflex OR enzymatic)<br>OR<br>TS=(fasciotom* OR aponeurotom* OR needle release OR PNF OR minimally invasive)<br>OR<br>TS=(fasciectom* OR aponeurectom*)))<br>Document type =(Article)
Database 4 name: CENTRAL

Search strategy:

(MeSH descriptor: [Dupuytren Contracture] explode all trees OR Dupuytren*: OR fibromatos*: OR fibroproliferative condition*: OR fibroproliferative disease*:)
AND
(MeSh descriptor: [Collagenases] explode all trees OR collagenase*: OR CCH : OR xiapex: OR xiaflex: OR enzymatic:
OR
(Mesh descriptor: [Fasciotomy] explode all trees OR fasciotom*: OR aponeurotom*: OR needle release: OR PNF: OR minimally invasive:)
OR
(fasciectom*: OR aponeurectom*:))

Appendix 2. Statistical analysis

We used a one stage-approach, which implicates a simultaneous analysis of IPD retrieved from eligible studies. In some studies, multiple fingers per patient were included. For these cases, we randomly selected one finger using the *dplyr* package in R (R Core Team, 2022; Wickham et al., 2022).

<u>Contracture correction</u>

We applied a linear mixed model with postoperative TED as the outcome. In addition to treatment (LF, PNF or CCH), we added age, preoperative TED, and whether it was a primary or repeated treatment as independent variables to the model to account for potential confounding effects. Because the residuals were non-normally distributed, we performed a square root transformation on the postoperative TED. Results were reported as regression coefficients, with corresponding 95% confidence intervals (CIs). Because transformed coefficients are hard to interpret, we also reported the estimated values of the postoperative TED for each treatment.

<u>Complications</u>

We applied a multinomial regression analysis with complication (none, mild or serious) as the outcome, and treatment, preoperative TED, age, primary or repeated treatment and the number of maximal potential complications (based on the number of complications being registered in the study) as independent variables in our model. Results were reported as ORs with corresponding 95% CIs.

Patient-reported hand function

We selected a linear mixed model, with the postoperative PROM score(s) as outcome, adding treatment, preoperative PROM score, age, primary or recurrent disease as explanatory variables to the model, and a random intercept to our model for the study.

Recurrence

We calculated the number and percentage (%) of recurrences and analysed the time to recurrence for each treatment. Since not all studies measured recurrence at the same time points, we applied survival analysis taking the follow-up times into account. Moreover, to take interval censoring into account, we applied a parametric interval-censored survival model with a lognormal distribution, which showed to have best model fit. We included treatment, age, sex, primary or recurrent disease as potential confounders. Patients for whom it was unknown whether they had had recurrence were excluded. Results were reported as regression coefficients and corresponding time ratios, which quantify the relative difference in time it takes until recurrence has occurred.

All studies were controlled for the potential clustering within a study, by adding a random intercept or stratum for the study the data originated from, and for the risk of bias (low, some concerns or high) and the study design (RCT or cohort). In addition, we planned to perform a sensitivity analysis on all outcomes by repeating the analyses, excluding studies with a high risk of bias. All analyses were performed in R version 4.1.3(R Core Team, 2022) and Rstudio version 2022.07.1(RStudio team, 2020), using the lme4(Bates et al., 2015), mclogit(Elff, 2022), survival(Thurneau, 2022) and flexsurv(Jackson, 2016) packages. A p-value of<0.05 was considered statistically significant.

Supplementary Figures

Online Figure 1: flow chart of assessing eligibility criteria for full text screening

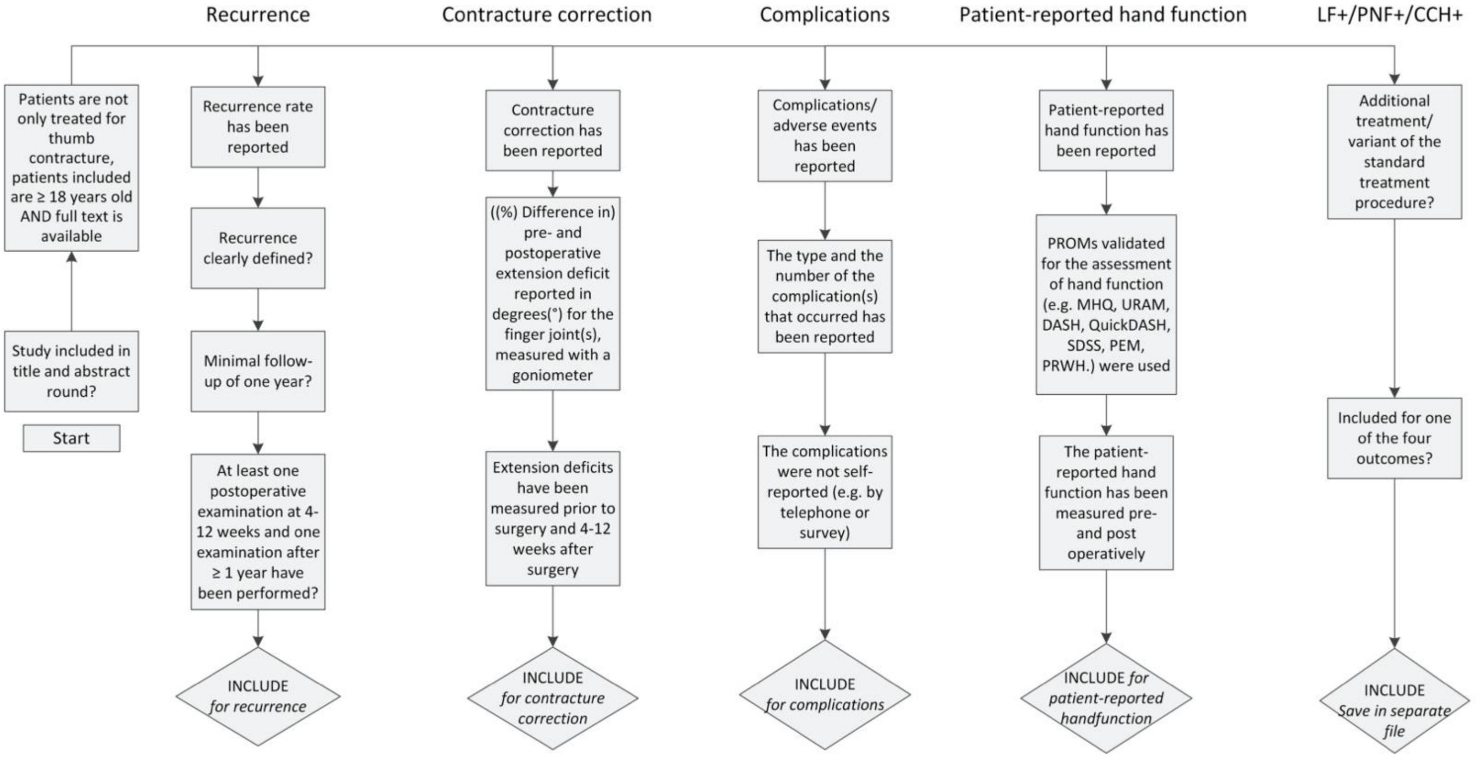

Abbreviations:

TPED:	Total passive extension deficit
TAED:	Total active extension deficit
LF:	Limited fasciectomy
PNF:	Percutaneous needle fasciotomy
CCH:	Collagenases clostridium histiolyticum
PROM:	Patient reported outcome measure
URAM:	Unité Rheumatologique des Affections de la Main
MHQ:	Michigan Hand Outcomes Questionnaire
DASH:	Disabilities of Arms, Shoulders and Hands questionnaire
SDSS:	South Hampton Dupuytren's Scoring scheme
PEM:	Patient Evaluation Measure
PRWH:	Patient Related Wrist and Hand evaluation

Online Figure 2: Predicted postoperative TED for patients treated for primary disease and patients treated for recurrent disease, based on the preoperative TED

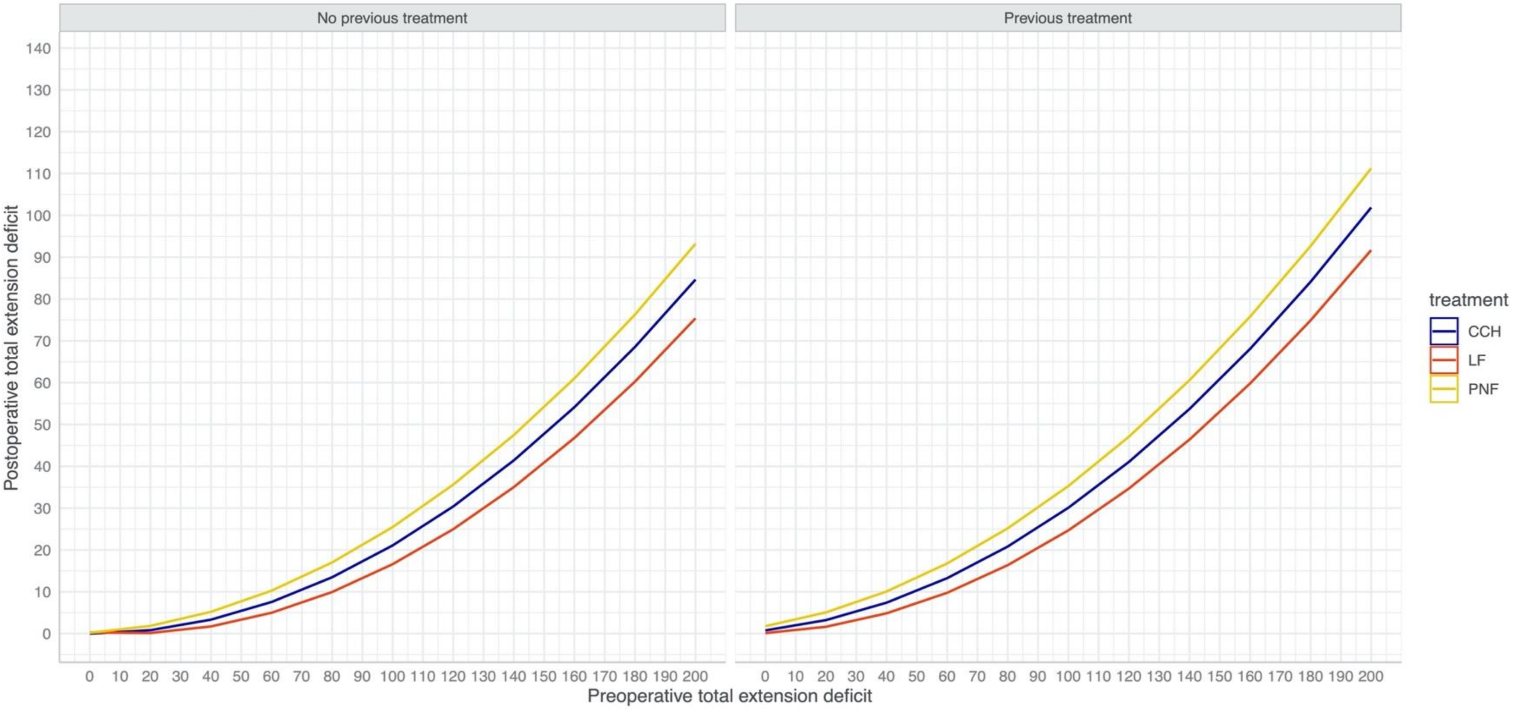

The x-axis represents the preoperative TED in degrees. The y-axis represents the postoperative TED in degrees. CCH=collagenase clostridium histolyticum; LF=limited fasciectomy; PNF=percutaneous needle fasciotomy.

Supplementary Tables

Online Table 1: Classification of complications between one week and three months after treatment for Dupuytren's disease

MILD	SERIOUS
Skin rupture/fissure	Infection
Hematoma	Tendon rupture
Edema	Nerve damage
Blood blister	Ligamentous damage
Lymphadenopathy	Nerve injury
Wound complication	Arterial injury
Local discomfort	CTS
Pain hand/wrist/arm	CRPS-I
Scarring	Division of tendon
Scar related	Loss of finger
Itching	Pain 3 months
Purpura/rash	Cold intolerance
Nausea arthralgia	
Myalgia	
Headache	
Dizziness	
Increased perspiration	
Thrombocytopenia	
Contusion	
Axillary tenderness	
Erythema	
Grip strength	
Wound dehiscence	
Skin healing	
Flexion deficit < 3 months	
Neuropraxia	
delayed wound healing	
Loss of flexion in operated finger<3 months	
Loss of flexion in other finger(s) <3 months	
Hypesthesia	
Anesthesia	
Paresthesia	
Sensory disturbance	
Sensory deficit	
Numbness	

CTS=carpal tunnel syndrome, CPRS-I=complex regional pain syndrome type I.

Online Table 2: Overview of the 211 studied eligible for at least one outcome

Authors	Year	Country	Design	Intervention	Splint	Outcome(s)	Centers	N	Start, end	Follow-up (m)	Funding/COI
Abdelrahman et al.	2020	SWE	Cohort$^{\alpha}$	PNF vs. CCH	Yes	C	1	157	2011, 2016	14 (SD 2.7)	NR/None
Abe	2020	JPN	RCT	PNF vs. CCH	No	A, C, P, R	1	72	2014, 2016	36	None/None
Abe et al.	2015	JPN	Self-controlled	PNF	Yes	A, C, R	1	51	2011, 2013	12	NR/None
Aguilella et al.	2022	ESP	Cohort	CCH, CCH+	Yes	A	1	90	NR	3	NR/None
Alberton et al.	2014	ITA	Uncontrolled	CCH	Yes	A, C	NR	40	2012, NR	6	Yes/None
Alencar et al.	2021	BRA	Uncontrolled	LF	No	C	1	247	2013, 2019	NA	None/None
Anwar et al.	2009	GBR	Uncontrolled	LF+	Yes	C	1	84	1990, 2004	14 (R 1.5-23)	NR/NR
Apard et al.	2011	FRA	Uncontrolled	LF+	No	C	1	27	2005, 2005	31	NR/None
Arora et al.	2016	AUT	Uncontrolled	CCH	Yes	C	1	120	NR	12	NR/None
Ashdown et al.	2021	GBR	Uncontrolled	LF	Yes	C	1	43	2013, 2015	69 (R 61-77)	None/None
Atroshi et al.	2014	SWE	Cohort	LF vs. CCH	Yes	A, C	1	32	2009, 2012	2 (IQR 1.5-3)	Yes/Yes
Atroshi et al.	2015	SWE	Cohort	CCH	Yes	C	1	146	2012, 2013	1	Yes/Yes
Aykut et al.	2017	TUR	Uncontrolled	LF	Yes	C	1	19	2006, 2014	48 (R 24-86)	NR/None
Badalamente et al.	2007	USA	RCT	CCH	Yes	A, C, R	1	35	NR	24	Yes/None
Badalamente et al.	2000	USA	Uncontrolled	CCH	Yes	C	1	35	NR	19$^{\delta}$	Yes/Yes
Badalamente et al.	2002	USA	RCT	CCH	Yes	C	2	80	NR	1	Yes/Yes
Bainbridge et al.	2012	CZE, DNK, FIN, FRA, DEU, HUN, ITA, NLD, POL, ESP, SWE, GBR	Cohort$^{\alpha}$	PNF vs. LF	Yes*	C	NR	3357	2008, 2008	NR	Yes/Yes
Bear et al.	2017	USA,AUS,SWE,GBR	Uncontrolled	CCH	Yes	A, C, R	12	52	2012, 2013	12	Yes/Yes
Beaudreuil et al.	2011	FRA	Self-controlled	PNF	NR	A, C	1	30	2005/2006	6	NR/None
Beaudreuil et al.	2011	FRA	Uncontrolled	PNF	NR	P	1	53	NR	1	Yes/Yes
Bekkering et al.	2015	NLD	Self-controlled	PNF	No	A, C, P	1	68	2011, 2013	1.5	Uncl./None
Bergovec et al.	2018	HRV	Self-controlled	LF	Yes	C, P	1	34	2000, 2006	114 (R 84-156)	None/None
Beyermann et al.	2004	DEU	Cohort	LF	Yes	C	1	43	NR	6	NR/NR
Binter et al.	2014	AUT	Self-controlled	CCH	Yes	A, C, R	1	37	NR	12	NR/None
Bismil et al.	2012	GBR	Uncontrolled	LF	No	C	NR	270	2009, 2012	1w	None/none
Bistué et al.	2018	ESP	Self-controlled	CCH	Yes	A, C, R	1	53	2012, 2016	12	NR/None
Blazar et al.	2016	USA	Self-controlled	LF+	Yes	C	1	19	NR	8 (95%CI 4-12)	None/Yes
Bowers et al.	2021	USA	RCT	CCH	Yes	A, P	1	29	2018, 2020	3	NR/None
Bozhanina et al.	2016	BGR	Self-controlled	PNF+	No	A, C, P	1	15	NR	1	NR/NR
Bradley et al.	2016	GBR	Self-controlled	CCH	NR	C, P	1	213	2011, 2014	25 (R 1-47)	None/None
Byström et al.	2022	SWE	RCT	PNF vs. LF	Yes*	P	1	156	2012, 2014	61 (R 58-76)	Yes/None
Chan et al.	2012	HKG	Self-controlled	LF	Yes	C	1	29	1999, 2008	32 (R 10-60)	NR/NR
Cheng et al.	2008	CHN	Uncontrolled	PNF	Yes	C	1	9	2002, 2005	22 (R 3-45)	NR/NR
Ciernik et al.	2021	DEU	Uncontrolled	LF+	No	C	1	6	2018, 2020	14 (R 6-30)	NR/None
Çirakli et al.	2013	TUR	Uncontrolled	LF	Yes	C	1	23	2005, 2013	NA	NR/NR
Citron et al.	2005	GBR	RCT	LF	Yes	C	1	100	1998, 2002	Uncl.	NR/NR

Coert et al.	2006	NLD	Self-controlled	LF	Yes*	C	1	261	1990, 1997	88	NR/NR
Authors	Year	Country	Design	Intervention	Splint	Outcome(s)	Centers	N	Start, end	Follow-up	Funding/COI
Coleman et al.	2014	AUS,USA	Self-controlled	CCH	Yes	A, C	8	60	2011, 2012	2	Yes/Yes
Coleman et al.	2012	AUS	Self-controlled	CCH	Yes	A, C	1	12	NR	3 (SD 0.25)	Yes/Yes
Considine et al.	2015	IRL	Self-controlled	CCH	No	A, C	1	10	NR	2 (±1)	NR/None
Cook et al.	2019	USA	Cohort	CCH	No	C	1	236	2010, 2017	0.5	None/Yes
Corain et al.	2019	ITA	Uncontrolled	CCH+	Yes	C	1	18	2014, 2016	14 (R 3-23)	None/None
Corain et al.	2020	ITA	Uncontrolled	CCH+	Yes	C	1	18	2014, 2016	14 (R 3-23)	None/None
Corrado et al.	2007	ITA	Self-controlled	PNF	NR	C	1	24	NR	NR	NR/NR
Costas et al.	2017	USA, AUS	RCT	CCH	No	C	11	75	2014, 2014	2	Yes/Yes
Coulibaly et al.	2020	SEN	Uncontrolled	PNF, LF	NR	C	1	26	2006, 2018	28 (R 14-72)	NR/None
Crivello et al.	2016	USA	Self-controlled	CCH	NR	A, C	NR	5	NR	1 (R 1–2)	Yes/None
David et al.	2020	GBR	Uncontrolled	CCH	Yes	A, C, R	1	292	NR	12	Yes/Yes
Davis et al.	2020	GBR	RCT	PNF vs. LF	Yes*	C, P	3	71	2015, 2016	6	Yes/None
DeVitis et al.	2020	ITA	Self-controlled	CCH	Yes	A, C, P, R	1	45	2012, NR	87 (±1)	None/None
Denkler	2005	USA	Cohort	LF	No	C	2	66	NR, NR	10	NR/NR
Denkler et al.	2021	USA	Self-controlled	CCH+	Yes	C	1	15	NR	6 (R 2-28)	None/Yes
Dhawan et al.	2019	CHN and/or USA	Self-controlled	CCH+	Yes	C, P, R	NR	35	2015, 2016	24	None/None
Dias et al.	2013	GBR	Cohort	LF, DF	Yes	A, P	1	63	NR	60	None/None
Donaldson et al.	2010	GBR	Cohort	LF	Yes	C	1	52	2006, 2007	6 (R 3-12)	NR/None
Eberlin et al.	2015	USA	Self-controlled	LF, CCH	Yes	C	1	11	2010, 2014	16 (R 2-29)	NR/None
Edmunds et al.	2011	AUS	Uncontrolled	LF, DF	Yes*	A, C	1	75	NR	2 (R 0.5-10)	None/None
Engstrand et al.	2014	SWE	Self-controlled	LF	Yes	A, C, P, R	1	90	NR	11[δ]	Yes/None
Fagernaes et al.	2018	DNK	Cohort	CCH vs. CCH	No	C	1	90	2012, 2014	3	None/None
Fei et al.	2019	USA	Self-controlled	CCH	Yes	A	1	21	2010, 2016	3	NR/Yes
Ferrari et al.	2020	FRA	Self-controlled	PNF	NR	A, C, P	1	30	2008, 2009	60	NR/None
Fletcher et al.	2019	AUS	Self-controlled	CCH	Yes	A, C, P	1	54	2016, 2017	7 [δ]	NR/None
Foucher et al.	2003	FRA	Self-controlled	PNF	Yes	C	1	211	NR	Uncl.	NR/NR
Foucher et al.	2001	FRA	Uncontrolled	PNF	Yes	C	1	NR	NR	NR	NR/NR
Ganeval et al.	2010	FRA	Self-controlled	PNF	Yes	C	1	20	2006, 2008	10 (R 3-24)	NR/NR
Garkisch et al.	2021	DEU	Self-controlled	LF	No	P	NR	23	2016, 2018	6	NR/Yes
Gaston et al.	2015	USA, AUS, NZL, DNK + Uncl.	Self-controlled	CCH	NR	A, C	56	715	2012, 2013	2	Yes/Yes
Giesberts et al.	2020	NLD	Self-controlled	LF	Yes	A	1	11	NR	3	Yes/NR
Gilpin et al.	2010	AUS	RCT	CCH	Yes	A, C, R	5	66	2007, 2008	12	Yes/Yes
Göransson et al.	2021	SWE	Uncontrolled	CCH	Yes	A, C, P	1	79	2012, 2014	60	None/None
Goubau et al.	2020	BEL	Cohort	CCH	NR	A, R	1	39	2014, 2016	27 (R 13-40)	None/None
Grandizio et al.	2017	USA	Uncontrolled	CCH	Yes	A, C	1	31	2014, 2016	1	None/None
Hansen et al.	2017	DNK	Cohort	CCH	Yes	C, P	1	340	2011, 2013	15	NR/None
Hartig-Andreasen et al.	2019	DNK	Uncontrolled	CCH	Yes	C	1	119	2013, 2016	30 (95%CI 12;49)	Yes/None
Hatta et al.	2019	JPN	Self-controlled	CCH	Yes	A, C, R	1	18	2015, 2016	24	NR/None
Hay et al.	2014	USA	Uncontrolled	LF	NR	C	NR	15	NA	NR	Yes/Yes
Hensler et al.	2020	CHE	Cohort	LF vs. CCH	NR	P	1	231	2013, 2017	12	NR/None

Herrera et al.	2015	USA	Self-controlled	PNF	Yes	C	1	182	2008, 2010	5 (R 0.25-28)	NR/None
Authors	Year	Country	Design	Intervention	Splint	Outcome(s)	Centers	N	Start, end	Follow-up	Funding/COI
Herrera et al.	2013	USA	Cohort[α]	PNF vs. LF	NR	C	1	48	2008, 2010	4	None/None
Hirata et al.	2017	JPN	Uncontrolled	CCH	Yes	A, C, R	30	102	2012, 2014	1	Yes/Yes
Hoang et al.	2019	USA	Cohort	LF+	NR	C	1	132	2007, 2017	19	NR/None
Horch et al.	2021	DEU	Cohort	LF+	NR	C, P	1	78	2003, 2012	NR	NR/None
Hurst et al.	2009	USA	RCT	CCH	Yes	A, C	16	308	2007, 2007	3	Yes/Yes
Hwee et al.	2017	USA	Uncontrolled	CCH	NR	C, R	1	113	2010, 2015	<60	None/Yes
Iwakawa et al.	2021	JPN	Uncontrolled	CCH	Yes	C	NR	10	2016, 2017	3	Yes/Yes
Johnston et al.	2008	GBR	Uncontrolled	LF	NR	A, P	1	19	NR	14 (R 11-16)	Yes/NR
Kaplan et al.	2015	USA	RCT	CCH	Yes	A, C, P	2	37	NR	3	Yes/Yes
Karakaplan et al.	2019	TUR	Uncontrolled	PNF	No	C	1	28	2011, 2015	29	None/None
Kasture et al.	2021	GBR	Uncontrolled	CCH	Yes	C	1	125	2013, 2016	61m (R 42-73)	None/None
Keller et al.	2017	AUT	Self-controlled	CCH	Yes	C	1	120	NR	12	Yes/None
Kemler et al.	2012	NLD	RCT	LF vs. LF	Yes	A, C, R	2	54	2007, 2008	12	None/None
Kitridis et al.	2019	GRC	Self-controlled	LF	Yes	C, P	1	30	2007, 2016	59 (R 24-132)	NR/None
Könneker et al.	2017	DEU	Uncontrolled	LF	Yes	C	1	16	NR	40 (SD 26)	None/None
Kuboi et al.	2021	JPN	Self-controlled	CCH	Yes	C	4	14	2019, 2020	6	NR/None
Lauritzson et al.	2017	SWE	Self-controlled	CCH	Yes	A, C, P, R	1	48	2011, 2012	26 (R 24-35)	None/Yes
Leclere et al.	2018	CHE	Cohort	LF vs. CCH	Yes	A, C, P	1	52	2012, 2013	24	NR/None
Leclere et al.	2014	CHE	Self-controlled	CCH+	Yes	C, P	1	33	2011, 2013	10 (SD 3)	NR/Yes
Lilly et al.	2010	USA	Cohort	LF+	NR	C	1	170	1992, 2006	8[δ]	None/None
Lyman et al.	2020	USA	Self-controlled	CCH	NR	A, P	3	109	2011, 2014	1	Yes/Yes
Malafa et al.	2016	USA	Self-controlled	CCH	Yes	A, C	1	36	Uncl.	6	NR/None
Manning et al.	2014	GBR	Self-controlled	CCH	Yes	C, P	1	45	2011, 2012	4	None/None
Mansha et al.	2017	GBR	Uncontrolled	PNF, LF	No	C	1	46	NR	15 (SD 10)	NR/None
Martín-Ferrero et al.	2013	ESP	Self-controlled	CCH	NR	A, C	1	35	2011, 2013	12	NR/None
Mavrogenis et al.	2009	GRC	Uncontrolled	LF	Yes	C	1	178	NR	79 (R24-108)	NR/NR
McFarlane et al.	2016	GBR	Uncontrolled	CCH	NR	P	NR	49	2012, 2012	24 (R 23-25)	NR/None
McMahon et al.	2013	USA	Self-controlled	CCH	Yes	C	1	102	2010, 2012	15 (R 6-25)	NR/Yes
McMillan et al.	2012	CAN	RCT	PNF vs. PNF+	Yes	A, C	1	47	NR	6	Yes/None
McMillan et al.	2014	CAN	RCT	PNF+	Yes	R	1	44	NR	7-53	Yes/Yes
Medjoub et al.	2014	GBR	Self-controlled	PNF	Yes	A, C	1	49	2008, 2010	18	None/None
Melamed et al.	2017	USA	case-control	LF	No	C	1	38	2004, 2009	62 (R 3-204)	None/None
Mendelaar et al.	2019	NLD	Cohort[γ]	LF, PNF, CCH, DF	NR	A, C, P	16	114	2011, 2017	3	None/None
Messana et al.	2021	ITA	RCT	CCH	NR	C	1	39	2019, 2019	1w	NR/None
Mi et al.	2021	CHN	Self-controlled	PNF+	NR	C, P	1	42	2014, 2019	27 (R 24-35)	None/None
Mickelson et al.	2014	USA	RCT	CCH	Yes	A, C	1	43	2012, 2013	1	None/None
Miranda et al.	2018	GBR	Cohort	LF+	Yes	C	1	585	2001, 2012	71[δ]	NR/None
Misra et al.	2007	GBR	Self-controlled	LF	Yes	C	1	37	1998, 2002	18 (R 12-36)	NR/None
Molenkamp et al.	2017	NLD	Uncontrolled	PNF	No	A, C, R	1	451	2004, 2014	NR	NR/Yes
Moog et al.	2019	DEU	Uncontrolled	PNF	Yes	C	NR	53	2008, 2018	31 (R 12-50)	None/None
Murphy et al.	2017	GBR	Self-controlled	CCH	Yes	A, P	1	20	NR	23 (R 17-27)	NR/NR
Naam	2013	USA	Cohort[β]	LF vs. CCH	Yes	C	1	46	NR	35[δ]	`Yes/Yes

Nayar et al.	2019	USA	Self-controlled	CCH	Yes	A, C, R	1	34	2010, 2014	24	NR/None
Authors	Year	Country	Design	Intervention	Splint	Outcome(s)	Centers	N	Start, end	Follow-up	Funding/COI
Nelson et al.	2010	CAN	Cohort	LF	Uncl.	C	2	111	2001, 2007	NR	None/None
Neuwirth et al.	2016	AUT	Cohort[α]	LF vs. CCH	Yes	C	1	40	2012, 2014	20 (R 3-44)	NR/None
Neuwirth et al.	2019	AUT	Uncontrolled	CCH	Yes	C	Uncl.	256	2011, 2018	10	NR/none
Noland et al.	2022	USA	Cohort	CCH	NR	C	1	197	2010, 2017	10 (R 0-72)	None/None
Nordenskjold et al.	2019	SWE	Self-controlled (RQ1); Cohort (RQ2)	CCH	Yes	A, C, R	1	86	2012, 2013	35*	Yes/Yes
Noureddine et al.	2020	GBR	Uncontrolled	LF	No	A, C, P	1	30	2017, 2018	3	None/None
Nydick et al.	2013	USA	Cohort[α]	PNF vs. CCH	Yes	C	1	59	NR	6 (R 3-28)	NR/Yes
Odinsson et al.	2016	NOR	Self-controlled	CCH	Yes	P	1	77	2012, 2013	12	NR/NR
Oreroglu et al.	2020	TUR	Self-controlled	PNF+	Yes	A, C, R	NR	30	2008, 2010	12	None/None
Ozkaya et al.	2010	TUR	Uncontrolled	LF	Yes	C	1	42	1995, 2005	60 (R 12-108)	NR/NR
Patel et al.	2021	GBR	Cohort	PNF	Yes	C	1	74	2017, 2020	12 (IQR 7–18)	None/None
Peimer et al.	2013	USA,GBR ,DNK,FIN ,SWE,AU S	Self-controlled	CCH	Yes	A, C, R	39	643	2009, 2013	60	Yes/Yes
Peimer et al.	2013	USA	Uncontrolled	CCH	Yes	C	10	463	2010, 2010	NR	Yes/Yes
Peimer et al.	2015	USA,GBR ,DNK,FIN ,SWE,AU S	Self-controlled	CCH	NR	R	39	644	2009, 2013	60	Yes/Yes
Pereira et al.	2012	PRT	Uncontrolled	PNF	Yes	C	1	36	2005, 2010	28 (R 12-63)	None/NR
Pérez-Giner et al.	2015	ESP	Uncontrolled	CCH	Yes	A	1	40	2011, 2014	3	NR/None
Pess et al.	2018	USA, AUS, NZL, DNK + ?	Self-controlled[β]	CCH	NR	A	56	346	2012, 2013	1	Yes/Yes
Pess et al.	2012	USA	Uncontrolled	PNF	Yes	C	NR	474	2005, 2008	NR (R 36-74)	NR/NR
Phan et al.	2021	AUS	Cohort	CCH	NR	P	1	184	2016, 2018	6	None/None
Pototschnig et al.	2017	DEU	Uncontrolled	CCH	Yes	A, C	1	78	2011, NR	6	NR/Yes
Radhamony et al.	2022	GBR	Uncontrolled	LF	Yes	A, C, R	3	130	2014, 2020	47	None/None
Rahr et al.	2011	DNK	Self-controlled	PNF	NR	A, C, R	1	149	2007, 2007	24	None/None
Reynolds et al.	2020	AUS	Self-controlled	CCH	Yes	A, C, P	1	82	2016, 2017	Uncl.	None/none
Ribak et al.	2013	BRA	Cohort	LF vs. PNF	Yes	A, C, R	NR	33	NR	12	NR/None
Rodrigues et al.	2017	GBR	Cross-sectional	PNF, LF, DF	NR	C	5	413	2011, 2013	60	Yes/None
Rodrigues et al.	2017	GBR	Cohort	LF,DF	NR	P	1	101	2011, 2013	12	Yes/None
Rohit et al.	2019	AUT	Uncontrolled	CCH	No	A, C	17	788	2011, 2017	12	Yes/None
Roulet et al.	2018	FRA	Self-controlled	LF+	Yes	C	1	56	1989, 1999	258 (R 224-316m)	NR/None
Roy et al.	2006	GBR	Uncontrolled	LF+	Yes	C	1	79	1990, 1998	53 (R 24-120)	None/NR
Sahemey et al.	2021	GBR	Cohort[β]	LF, PNF, CCH	Yes	C	1	151	2014, 2018	76 (IQR 70-82)	None/None
Sakai et al.	2019	JPN	Cohort	CCH	Yes	A, C	1	28	2015, 2017	3	None/None
Sakellariou et al.	2015	USA	Uncontrolled	PNF+	Yes	C	1	48	NR	26 (R 12-32)	NR/Yes
Sambuy et al.	2020	BRA	RCT	LF, LF+	Yes	A, C, P	1	50	2014, 2017	12	NR/None
Sanjuan-Cervero et al.	2018	ESP	Cohort	CCH	No	A	1	157	NR	1	NR/None
Sanjuan-Cervero et al.	2018	ESP	Cohort	CCH	NR	C	1	215	2011, 2016	1	None/Yes
Sanjuan-Cervero et al.	2018	ESP	Cohort	CCH	NR	C	1	151	2011, 2017	1	None/None

Authors	Year	Country	Design	Intervention	Splint	Outcome(s)	Centers	N	Start, end	Follow-up	Funding/COI
Sanjuán-Cerveró et al.	2018	ESP	Self-controlled	CCH	NR	A, C, P, R	1	51	20112017	14 (95%CI 13-15)	NR/None
Scheibler et al.	2019	CHE	Self-controlled	CCH	Yes	P	1	92	2012, 2016	12	None/Yes
Scherman et al.	2016	SWE	RCT	PNF vs. CCH	Yes*	A, C, P, R	2	93	2012, 2013	12	None/Yes
Scherman et al.	2018	SWE	RCT	PNF vs. CCH	Yes*	A, P, R	2	93	2012, 2013	36	Yes/Yes
Shewring et al.	2014	GBR	Uncontrolled	LF	NR	C	1	20	NR	NA	None/None
Simon-Perez et al.	2018	ESP	Uncontrolled	CCH	Yes*	C	1	71	2011, 2013	>48	NR/None
Skirven et al.	2013	USA	Self-controlled	CCH+	Yes	A, C	1	21	NR	1	Yes/Yes
Skoff	2004	USA	Cohort	LF+	Yes	C, P	1	30	NR;NR	36$^{\delta}$	NR/NR
Skov et al.	2017	DNK	RCT	PNF vs. CCH	Yes	A, C, R	1	50	2012, 2013	24	NR/Yes
Sood et al.	2014	USA	Self-controlled	CCH	Yes	C	1	16	2010, 2013	12	NR/NR
Sorene et al.	2007	ISR	Uncontrolled	LF	NR	C	1	19	1995, 2005	30 (R 12-118)	NR/NR
Spanholtz et al.	2011	DEU	Uncontrolled	CCH	Yes	C	1	8	NR	0.5	NR/None
Spies et al.	2016	DEU	Uncontrolled	PNF	Yes	C	NR	15	NR	40	Yes/None
Spies et al.	2016	DEU	Uncontrolled	LF	NR	C	1	18	NR	94 (R 70-114)	NR/None
Stahl et al.	2008	ISR	Uncontrolled	LF	NR	C	1	48	1986, 2005	30 (R 18-228)	NR/NR
Stromberg et al.	2016	SWE	RCT	PNF vs. CCH	Yes*	C, P	1	140	2012, 2014	12	Yes/None
Stromberg et al.	2017	SWE	RCT$^{\beta}$	PNF vs. CCH	NR	C	1	39	2013, 2014	12	Yes/None
Stromberg et al.	2018	SWE	RCT	PNF vs. CCH	Yes*	P	1	156	2012, 2014	24	Yes/None
Syed et al.	2014	GBR	Self-controlled	CCH	No	A, C, P, R	1	56	2012, 2013	5$^{\delta}$	NR/None
Terry et al.	2014	USA	Uncl.	LF	NR	C	1	43	2005, 2012	22	None/None
Therkelsen et al.	2020	DNK	Uncontrolled	PNF	NR	C	1	2257	2007, 2015	86 (IQR 59-114)	Yes/None
Thoma et al.	2014	CAN	Self-controlled	LF	NR	P	3	33	2007, 2010	12	None/None
Titarenko	2009	RUS	Uncontrolled	LF	NR	C	1	277	1997, 2006	NR	NR/NR
Trâmbiţaş et al.	2021	ROU	Cohort	PNF	Yes	C	1	40	2017, 2020	12	None/None
Tripoli et al.	2008	FRA, LUX	Uncontrolled	LF+	Yes	C	2	98	2001, 2006	13	NR/NR
Tripoli et al.	2010	ITA	Uncontrolled	LF+	Yes	C	1	15	2006, 2009	22 (R 4-36)	None/NR
Trybus et al.	2019	POL	Self-controlled	LF	NR	A, P	1	95	2006, 2011	12	NR/NR
Tuncel et al.	2017	TUR	Self-controlled	PNF+	Yes	A, C	NR	17	NR	12	NR/None
Ullah et al.	2009	GBR	RCT	LF	No	A, C, P	1	79	NR	36	NR/None
VanBeeck et al.	2017	BEL	Uncontrolled	CCH	Yes	A, R	1	87	2011, 2012	<24	NR/Yes
vanRijssen et al.	2006	NLD	RCT	PNF vs. LF	No	A, C, P	1	113	2002, 2005	1.5	NR/None
vanRijssen et al.	2012	NLD	RCT	PNF vs. LF	No	A, R	1	113	2002, 2005	60	NR/None
VanRijssen et al.	2006	NLD	Self-controlled	PNF	No	C	1	52	2001, 2005	33 (SD 13)	NR/NR
Verheyden	2015	USA	Uncontrolled	CCH	Yes*	A, C	1	144	2010, 2012	2	None/Yes
Verstreken et al.	2016	BEL	Self-controlled	CCH	Yes*	A, C, P	9	104	2013, 2014	1	None/Yes
Villanueva et al.	2022	ESP	Uncontrolled	PNF+	Yes*	C, P	NR	35	2014, 2018	24 (R 12-48)	None/None
Vollbach et al.	2013	DEU	Cohort	LF vs. CCH	NR	A, C, P, R	1	27	20112011	12	NR/None
Warwick et al.	2015	DNK, FRA, DEU, HUN, ITA, ESP, SWE, GBR	Self-controlled	CCH	NR	A, C, P	28	254	2010, 2012	6	Yes/Yes
Warwick et al.	2016	GBR	Uncontrolled	CCH	NR	C	1	237	2011, 2014	NA	None/Yes
Waters et al.	2015	Uncl.	Uncontrolled	CCH	Yes	C	2	8	2010, 2011	7 (R 1-22)	NR/None

Watt et al.	2010	USA	Uncontrolled$^{\beta}$	CCH	Yes	A	1	8	1999, 2000	96	Yes/Yes
Authors	Year	Country	Design	Intervention	Splint	Outcome(s)	Centers	N	Start, end	Follow-up	Funding/COI
Wehrli et al.	2016	CHE	Cohort	LF vs. CCH	Yes	P	1	57	2013, 2014	12	NR/None
Wei et al.	2020	USA	Cohort	CCH	Yes	A	1	117	2012, 2018	1	NR/Yes
Wei et al.	2015	Uncl.	Cohort	CCH, LF	Yes	C	1	37	2002, 2005	>24	NR/NR
Werlinrud et al.	2018	DNK	Cohort	CCH	Yes	P	1	104	2011, 2012	56$^{\delta}$	None/None
White et al.	2012	GBR	Uncontrolled	LF+	Yes	C	2	27	1999, 2004	21	None/None
Wiseman et al.	2019	AUS	Self-controlled	CCH	No	A, C	1	137	2014, 2017	1	None/None
Witthaut et al.	2013	USA, AUS, GBR, CHE, SWE, DNK, FIN	Uncontrolled	CCH	Yes	A, C, R	34	587	2007, 2008	9	Yes/Yes
Zachrisson et al.	2020	SWE	Self-controlled	PNF	Yes*	A, C, P, R	1	42	2010, 2012	78 (IQR 55)	Yes/None
Zhao et al.	2019	USA	Self-controlled	CCH	NR	A, C	7	100	2010, 2015	Uncl.	None/None
Zhou et al.	2015	NLD	Cohort	LF vs. CCH	Uncl.	A, C, P	7	132	2011, 2014	3 (R 1.5-3)	Yes/None
Zhou et al.	2016	NLD	Cohort	PNF vs. LF	Yes	A, C, P	6	181	2011, 2014	3	None/None
Zhou et al.	2016	NLD	Cohort	LF	NR	A, C	6	194	Uncl.	10 (R 6-12)	Yes/None
Zoubos et al.	2014	GRC	Self-controlled	LF+	Yes	C	1	31	1990, 2009	25 (R 12-35)	NR/NR

In randomized controlled trials, patients are randomly allocated to a treatment and the outcomes between the two groups are compared. In cohort studies, patients from two or more treatment groups are observed over a certain period and the outcomes are compared. In self-controlled studies, the treatment outcomes before and after treatment in one single population (i.e., before-after design) are compared and analyzed. In uncontrolled studies, only the results of a treatment in one study population are reported. α=the outcome complications did not fulfill the criterium: 'outcome data prospectively collected, for the purpose of this study'; β=data overlap with another study; γ=no comparison between LF, PNF and CCH; δ=weighted mean calculated; *=in specific cases. LF+/PNF+/CCH+: additional treatment or variant of the standard procedure (e.g., PNF + lipofilling, or LF + radiotherapy); RCT=randomized controlled trial; R=range; IQR=interquartile range; m=months; A=angular deformity correction; C=complications; P=PROMs; R=recurrence. y.; Uncl.=unclear; COI=conflict of interest; NR=not reported; Uncl.=unclear. Countries are abbreviated according to the ISO 3166 country codes.(International Organization for Standardization (ISO), 2006)

Online Table 3: Results of the risk of bias assessment

ROB-2 tool for randomized controlled trials

Study	Experimental	Comparator	Outcome	D1	D2	D3	D4	D5	Overall
Abe 2020	PNF	CCH	Complications	+	+	+	!	!	!
Abe 2020	PNF	CCH	PROM	+	!	+	!	!	!
Abe 2020	PNF	CCH	Recurrence	+	!	+	!	!	!
Davis 2020	PNF	LF	Contraction correction	+	+	+	+	+	+
Davis 2020	PNF	LF	Complications	+	+	+	!	+	!
Davis 2020	PNF	LF	PROMs	+	+	-	!	+	-
Scherman 2016&2018	PNF	CCH	Contraction correction	+	!	+	!	!	!
Scherman 2016&2018	PNF	CCH	Complications	+	!	+	!	!	!
Scherman 2016&2018	PNF	CCH	PROMs	+	!	+	+	!	!
Scherman 2016&2018	PNF	CCH	Recurrence	+	!	+	!	!	!
Skov 2017	PNF	CCH	Contraction correction	!	+	+	!	!	!
Skov 2017	PNF	CCH	Complications	!	+	+	!	!	!
Skov 2017	PNF	CCH	Recurrence	!	+	+	!	!	!
Stromberg 2016	PNF	CCH	Complications	!	+	+	!	!	!
Stromberg 2016	PNF	CCH	PROM	!	+	+	+	!	!
Van Rijssen 2006&2012	PNF	LF	Contraction correction	+	!	+	!	!	!
Van Rijssen 2006&2012	PNF	LF	Complications	+	!	+	!	!	!
Van Rijssen 2006&2012	PNF	LF	PROMs	+	!	+	!	!	!
Van Rijssen 2006&2012	PNF	LF	Recurrence	+	+	-	!	!	-

ROBINS-I tool for non-randomized studies of interventions

Study	Experimental	Comparator	Outcome	D1	D2	D3	D4	D5	D6	D7	Overall
Atroshi 2014	CCH	LF	Contraction correction	-	+	+	+	?	-	!	-
Leclere 2018	CCH	LF	Contraction correction	-	+	+	+	?	!	!	-
Leclere 2018	CCH	LF	PROMs	-	+	+	+	?	!	!	-
Ribak 2013	PNF	LF	Contraction correction	-	+	+	+	?	?	!	-
Ribak 2013	PNF	LF	Recurrence	-	+	+	+	?	?	!	-
Vollbach 2013	CCH	LF	Contraction correction	-	+	+	?	?	!	!	-
Vollbach 2013	CCH	LF	Complications	-	+	+	?	?	!	!	-
Vollbach 2013	CCH	LF	PROMS	-	+	+	?	?	!	!	-
Vollbach 2013	CCH	LF	Recurrence	-	+	+	?	?	-	!	-
Wei 2015	CCH	LF	Complications	-	+	+	+	?	!	?	-
Zhou 2015	CCH	LF	Contraction correction	!	+	+	+	!	!	!	!
Zhou 2015	CCH	LF	Complications	!	+	+	+	?	!	!	!
Zhou 2015	CCH	LF	PROMs	!	+	+	+	?	!	!	!
Zhou 2016	PNF	LF	Contraction correction	!	+	+	+	+	!	!	!
Zhou 2016	PNF	LF	Complications	!	+	+	+	+	!	!	!
Zhou 2016	PNF	LF	PROMs	!	+	+	+	-	!	!	-

Symbol	Meaning	ROB-2		ROBINS-I	
!	Some concerns	D1	Randomisation process	D1	Bias due to confounding
-	High risk	D2	Deviations from intended interventions	D2	Bias due to selection of participants
+	Low risk	D3	Missing outcome data	D3	Bias in classification of interventions
?	No information	D4	Measurement of the outcome	D4	Bias due to deviations from intended interventions
		D5	Selection of the reported result	D5	Bias due to missing data
				D6	Bias in measurement of outcomes
				D7	Bias in selection of the reported result

D1, domain one; D2, domain two, etc.

PNF=percutaneous needle fasciotomy; CCH=collagenase clostridium histolyticum; TED=total extension deficit; RCT=randomized

controlled trial. D1=domain one, D2=domain two, etc.

Online Table 4: Predicted postoperative TED [95%CI], adjusted for preoperative TED, primary or recurrent disease and treatment

	CCH		LF		PNF	
PREOP. TED	PRIMARY	RECURRENT	PRIMARY	RECURRENT	PRIMARY	RECURRENT
20°	1° [0, 3]	3° [1, 8]	0° [0, 1]	0° [0, 5]	2° [0, 5]	5° [2, 11]
40°	3° [1, 7]	7° [3, 13]	2° [0, 4]	5° [2, 10]	5° [2, 10]	10° [5, 17]
60°	8° [4, 12]	13° [7, 21]	5° [2, 9]	10° [5, 16]	10° [6, 16]	17° [10, 26]
80°	13° [8, 20]	21° [13, 30]	10° [6, 15]	16° [10, 24]	17° [11, 24]	25° [16, 36]
100°	21° [15, 29]	30° [21, 41]	17° [11, 24]	25° [17, 34]	25° [18, 34]	35° [25, 48]
120°	30° [22, 40]	41° [30, 54]	25° [18, 34]	35° [25, 46]	36° [26, 46]	47° [35, 61]
140°	41° [31, 53]	54° [40, 69]	35° [26, 46]	46° [34, 60]	47° [36, 60]	61° [46, 77]
160°	54° [42, 68]	68° [52, 86]	47° [35, 60]	60° [45, 76]	61° [47, 76]	76° [59, 95]
180°	69° [54, 85]	84° [66, 105]	60° [46, 76]	75° [58, 94]	76° [60, 95]	93° [73, 115]
200°	85° [67, 105]	102° [80, 126]	75° [59, 94]	92° [72, 114]	93° [74, 115]	111° [88, 137]

Adjusted for: age=65; ROB=high; design=cohort, study=0 (population-level). Preop.=preoperative; TED=total extension deficit; CCH=collagenase clostridium histolyticum; LF=limited fasciectomy; PNF=percutaneous needle fasciotomy

Online Table 5: Results from the linear mixed effects model on the postoperative TED including treatment with LF, PNF or CCH, age, previous treatment (y/n), preoperative TED study design, and risk of bias as independent variables

	COEFFICIENT	95% CI	P-VALUE	INTERPRETATION
Intercept (ref: CCH)	0.17	-1.30; 1.63	0.823	
PNF	0.45	0.05; 0.86	**0.028***	PNF resulted in a larger postoperative TED than CCH
LF	-0.52	- 0.93; -0.10	**0.015***	LF resulted in a smaller postoperative TED than CCH
Intercept (ref: LF)	-0.35	-1.83; 1.13	0.643	
PNF	0.97	0.59; 1.36;	**<0.001***	PNF resulted in a larger postoperative TED than LF
Age	-0.00	-0.02; 0.02	0.758	Age was not associated with postoperative TED
Previous treatment (ref: no)	0.89	0.39; 1.39	**0.001***	Treatment for recurrent disease was associated with a larger postoperative TED than for primary disease
Preoperative TED	0.05	0.04; 0.05	**<0.001***	A larger preoperative TED was associated with a larger postoperative TED
Study design (ref: cohort)	-1.00	-1.54; -0.46	**<0.001***	RCTs were associated with smaller postoperative TED than cohort studies
Risk of bias (ref: high)				
Low	1.73	0.62; 2.83	**0.002***	Low risk of bias was associated with a larger postoperative TED than high risk of bias
Some concerns	1.14	0.27; 2.01	**0.010***	Some concerns on bias was associated with a larger postoperative TED than high risk of bias

Coefficients represents the regression coefficient in degrees after square root transformation. *=statistically significant effect; LF=limited fasciectomy; PNF=percutaneous needle fasciotomy; CCH=collagenase clostridium histolyticum; TED=total extension deficit; RCT=randomized controlled trial

Online Table 6: Number of each serious complication, stratified by treatment

	CCH	LF	PNF
Serious complication, total (%)	9/244 (3.7)	7/147 (4.8)	8/263 (3.0)
Infection	0	2	1
Tendon rupture	0	0	0
Nerve injury	0	1	1
Ligamentous damage	0	0	0
Paresthesia/nerve damage	1	0	0
Arterial injury	0	0	0
CTS	0	1	0
CRPS-I	1	0	0
Tendon division	0	0	0
Loss of finger	0	0	0
Pain at three months	7	0	7
Cold intolerance	0	3	0

One patient in the PNF group had 2 serious complications: infection and pain at three months.
nCTS = carpal tunnel syndrome; CRPS-I = complex regional pain syndrome type 1.

Online Table 7: Results from the multinomial regression model on complications after treatment with LF, PNF or CCH

	COEFFICIENT	95% CI	P-VALUE	INTERPRETATION
Mild complications				
Intercept (ref: CCH)	-2.60	-5.67; 0.46	0.096	
PNF	-3.06	2.51; 3.61	**<0.001***	The odds of a mild complication is 21.4 (95%CI 12.4; 37.2) times lower when treated with PNF compared to CCH
LF	-2.51	-3.11; -1.92	**<0.001***	The odds of a mild complication is 12.4 (95%CI 6.8; 22.5) times lower when treated with LF compared to CCH
Intercept (ref: LF)	-5.12	-8.23; -2.01	**0.001**	
PNF	-0.55	-1.21; 0.10	0.099	No significant difference in mild complications between PNF and LF
Age	0.00	-0.02; 0.03	0.748	Age was not associated with mild complications
Previous treatment (ref: no)	0.16	-0.50; 0.82	0.633	Treatment for primary of recurrent disease was not associated with mild complications
Preoperative TED	0.01	0.00; 0.02	**0.047***	1° increase of preoperative TED increases the odds of a mild complication 1.01 (95%CI 1.00; 1.02) times
Number of registered complications	0.12	0.03;0.22	**0.009***	Each unit increase in the number of complications registered increases the odds of a mild complication 1.13 (95%CI 1.03; 1.25) times
Study design (ref: cohort)	1.54	-0.59; 3.68	0.157	Study design was not associated with mild complications
Serious complications				
Intercept (ref: CCH)	-7.17	-15.12; 0.78	0.077	
PNF	-0.97	-2.04; 0.09	0.072	No significant difference in serious complications between PNF and CCH
LF	0.34	-1.25; 1.93	0.675	No significant difference in serious complications between LF and CCH
Intercept (ref: LF)				
PNF	-1.31	-2.88; 0.26	0.101	No significant difference in serious complications between PNF and LF
Age	0.01	-0.04; 0.07	0.600	Age was not associated with serious complications
Previous treatment (ref: no)	1.51	0.06; 2.96	**0.042***	Treatment for recurrent disease resulted in a 5.6 (95%CI 1.1; 19.8) times higher odds of serious complications
Preoperative TED	0.00	-0.01; 0.02	0.554	Preoperative TED was not associated with serious complications
Number of registered complications	0.10	-0.15; 0.34	0.435	The number of complications registered was not associated with serious complications
Study design (ref: cohort)	1.60	-3.86; 7.06	0.566	Study design was not associated with serious complications

Risk of bias was not included as an independent variable in this analysis, because all included studies had some concerns of bias for this outcome. *=statistically significant effect; LF=limited fasciectomy; PNF=percutaneous needle fasciotomy; CCH=collagenase clostridium histolyticum; TED=total extension deficit; RCT=randomized controlled trial

Online Table 8: Available IPD and aggregate data for each PROM

PNF vs. CCH	URAM	MHQ	briefMHQ	DASH	quickDASH	PEM
Stromberg 2016+2018	2w,3m,12 m				2w, 3m, 12 m	
Scherman 2016+2018	3 m, 12m, 36m*				3 m, 12m, 36m	
Abe 2020	1m, 36m*					
PNF vs. LF						
Davis 2020	6m			6m		6m
Van Rijssen 2006+2012				1, 2, 3, 4, 5w		
Zhou 2016		6w-3m				
CCH vs. LF						
Zhou 2015		6w-3m				
Leclere 2018		24m				
Vollbach 2013		1m, 6m, 12m		1m, 6m, 12m		
Wehrli 2016			12m			
Hensler 2020	12 m		12 m			

IPD received is indicated in green. No IPD received but aggregate data available from full text article is indicated in red.*=no SD reported The time of the study measurements are indicated for each study. PROM=patient reported outcome measure; IPD=individual patient data; m=months, w=weeks. LF=limited fasciectomy; PNF=percutaneous needle fasciotomy; CCH=collagenase clostridium histolyticum; URAM=Unité Rhumatologique des Affections de la Main; MHQ=Michigan Hand Questionnaire; DASH=Disabilities of the Arm, Shoulder and Hand; PEM=patient evaluation measure

Online Table 9: Results of the parametric interval-censored survival analysis for time to recurrence

	COEFFICIENT	95% CI	P-VALUE	INTERPRETATION
Intercept (ref: CCH)	*1.26*	-0.67; 3.18	0.202	
PNF	-0.13	-0.52; 0.27	0.532	No significant difference in time to recurrence between PNF and CCH
LF	1.75	0.51; 2.99	**0.006***	Recurrence occurred 6.5 (95%CI 2.0; 21.3) times later after LF than after CCH
Intercept (ref: LF)	3.01	0.87; 5.15	**0.006**	
PNF	-1.88	-3.06; -0.70	**0.002***	Recurrence occurred 5.8 (95%CI 1.7; 20.0) times earlier in PNF than in LF
Age	0.04	0.01; 0.06	**0.003***	A one-year increase in age resulted in 1.04 (95%CI 1.01; 1.07) times longer time to recurrence per year
Sex (ref: female)	0.54	0.04; 1.04	**0.035***	Being male resulted in 1.7 (95%CI 1.0; 2.8) times longer time to recurrence than being female
Risk of bias (ref: high)	-0.57	-1.41; 0.27	0.182	Risk of bias was not associated with time to recurrence
Study (stratum)†				
Scherman 2018	-0.26	-0.56; 0.04	0.088	Scherman 2018 was not associated with time to recurrence
Skov 2017	0.36	0.01; 0.72	**0.044***	Skov 2017 resulted in 1.4 (95%CI 1.0; 2.0) times longer time to recurrence
Van Rijssen 2012	0.56	0.09; 1.03	**0.020***	Van Rijssen 2012 resulted in 1.8 (96%CI 1.1; 2.8) times longer time to recurrence

Study design was not included as an independent variable in this analysis, because all included studies for this outcome were RCTs. All patients were treated for primary Dupuytren's disease, so this variable was not included in the analysis.
*=statistically significant effect; LF=limited fasciectomy; PNF=percutaneous needle fasciotomy; CCH=collagenase clostridium histolyticum; TED=total extension deficit; RCT=randomized controlled trial. †The effect estimates of the stratum reflect the results of the survival model for each subpopulation (i.e., study).

REFERENCES

Bates, D., Mächler, M., Bolker, B., & Walker, S. (2015). Fitting Linear Mixed-Effects Models Using lme4. *Journal of Statistical Software*, *67*(1 SE-Articles), 1–48. https://doi.org/10.18637/jss.v067.i01

Elff, M. (2022). *mclogit: Multinomial Logit Models, with or without Random Effects or Overdispersion. R package version 0.9.4.2.* https://cran.r-project.org/web/packages/mclogit/mclogit.pdf

International Organization for Standardization (ISO). (2006). *Reserved code elements under ISO 3166-1 "Codes for the representation of names of countries and their subdivisions – Part 1: Country codes."* https://www.iso.org/obp/ui/#search/code/

Jackson, C. H. (2016). flexsurv: A Platform for Parametric Survival Modeling in R. *Journal of Statistical Software*, *70*. https://doi.org/10.18637/JSS.V070.I08

R Core Team. (2022). *R: A language and environment for statistical computing.* (4.1.3). R Foundation for Statistical Computing, Vienna, Austria. http://www.r-project.org/index.html

RStudio team. (2020). *RStudio: Integrated Development for R* (2022.07.1). Posit PBC. www.rstudio.com/.

Thurneau, T. (2022). *A Package for Survival Analysis in R. R package version 3.3-1*. https://cran.r-project.org/package=survival%3E.

Wickham, H., François, R., Henry, L., & Müller, K. (2022). *dplyr: A Grammar of Data Manipulation. R package version 1.0.8.* https://dplyr.tidyverse.org/reference/index.html
